# Mediators of the Association Between Child Education and Adult Health

**DOI:** 10.1001/jamanetworkopen.2025.8855

**Published:** 2025-05-23

**Authors:** Maria E. Bleil, Glenn I. Roisman, Deven T. Hamilton, Steven E. Gregorich, Sophia W. Magro, Bradley M. Appelhans, Cathryn Booth-LaForce, Robert C. Pianta

**Affiliations:** 1 Child, Family, & Population Health Nursing, University of Washington, Seattle; 2 Institute of Child Development, University of Minnesota, Minneapolis; 3 Center for Studies in Demography & Ecology, University of Washington, Seattle; 4 Department of Medicine, University of California, San Francisco; 5 Institute for Innovations in Developmental Sciences, Northwestern University, Chicago, Illinois; 6 Department of Family and Preventive Medicine, Rush University Medical Center, Chicago, Illinois; 7 School of Education & Human Development, University of Virginia, Charlottesville

## Abstract

**Importance:**

Educational attainment is a key social determinant of cardiometabolic disease in adulthood, yet knowledge about the pathways through which educational experiences in childhood may shape cardiometabolic health is limited.

**Objective:**

To explore whether associations between educational experiences in childhood and cardiometabolic health in adulthood are mediated by adult socioeconomic status and health behaviors.

**Design, Setting, and Participants:**

Participants were from the Eunice Kennedy Shriver National Institute of Child Health and Human Development (NICHD) Study of Early Child Care and Youth Development (SECCYD), a birth cohort (1991-2009) recruited to participate in a 30-year assessment (ages 26-31) between January 2018 and December 2022 focused on cardiometabolic health outcomes.

**Exposures:**

Exposures were child educational indicators (student social competence, student-teacher relationship quality, classroom emotional and instructional quality, and academic achievement), and adult pathways were mediators (income, educational level, diet quality, activity level, sleep duration, and smoking status).

**Main outcomes and Measures:**

The primary outcome was a composite measure of metabolic syndrome, in which assessments of waist circumference, systolic and diastolic blood pressure, levels of hemoglobin A_1c_, C-reactive protein, and high-density lipoprotein were combined to form a single unit-standardized index of cardiometabolic risk (CMR).

**Results:**

Among 700 participants (374 female [53.4%]; mean [SD] age, 28.6 [1.2] years; 70 [10.0%] Black, 45 [6.4%] Latino, 552 [78.9%] White, and 33 [4.7%] other), linear regression models supported the role of adult income and adult diet quality as mediators. Independent of covariates (sex, race and ethnicity, child body mass index percentile, and parental socioeconomic status), adult income mediated associations between indicators, including student social competence (*b* = −0.001; 95% CI, −0.002 to −0.0003; *P* = .02), student-teacher relationship quality (*b* = −0.002; 95% CI, −0.004 to −0.001; *P* = .02), classroom instructional quality (*b* = −0.012; 95% CI, −0.034 to −0.001; *P* = .06 for trend), and academic achievement (*b* = −0.001; 95% CI, −0.002 to −0.0004; *P* = .02), and adult CMR. Adult diet quality mediated associations between indicators, including student social competence (*b* = −0.002; 95% CI, −0.004 to −0.001; *P* < .001) and student-teacher relationship quality (*b* = −0.003; 95% CI, −0.005 to −0.001; *P* = .01), and adult CMR. Results for the other candidate mediators were nonsignificant.

**Conclusions and Relevance:**

In this birth cohort study, results showed that associations between educational experiences in childhood and cardiometabolic health in adulthood were mediated by adult income for 4 of 5 indicators and by adult diet for 2 of 5 indicators. This suggests that, across diverse educational experiences, long-term health benefits may be transmitted through having a higher adult income and a healthier adult diet. Findings offer a developmental framework for understanding the well-established education-health gradient, highlighting opportunities for early health promotion.

## Key Points

**Question:**

Are associations between child education and adult health mediated by adult socioeconomic status and health behaviors?

**Findings:**

In a birth cohort study of 700 participants, results showed associations between educational experiences in childhood and cardiometabolic health in adulthood were mediated by adult income for 4 of 5 educational indicators and by adult diet for 2 of 5 educational indicators, suggesting long-term health benefits may be transmitted through having a higher adult income and a healthier adult diet.

**Meaning:**

Results of this study suggest that early academic and socioemotional skills might be targeted for augmentation in school and nonschool settings to protect long-term cardiometabolic health.

## Teaser

This cohort study examines potential mediators of life course associations between early education and adult cardiometabolic health in a birth cohort of adults undergoing 30-year follow-up.

## Introduction

The seminal work of Adler et al^1,2,3,4,5,6^ has shaped decades of research focused on the social determinants of health and the role of social factors in perpetuating disparities in health outcomes.^7,8,9,10^ As a result of this research, it is now widely accepted that socioeconomic status (SES) is a powerful predictor of health and that associations between SES and health are graded, reflecting increasing health benefits across increasing social strata.^11,12,13,14,15,16^ Underpinning SES, educational status has been shown to have heath protective effects that are large in magnitude, persist over the life course, and are present across sex, race and ethnicity, geography, and multiple health outcomes,^17^ including all-cause and cardiovascular mortality, cardiovascular risk factors, type 2 diabetes, obesity, and inflammation.^18,19,20,21,22,23^ Yet, even with this knowledge, it has been difficult to harness interventions that are effective in disrupting existing social structures or in supporting vulnerable individuals. In fact, compared with other high-income countries, the US has the poorest standing with respect to life expectancy, infant mortality, obesity prevalence, and health care spending.^24,25,26^ All the while, gaps in health outcomes between the privileged and the less fortunate groups continue to grow.^27,28,29,30^

Studies of the education-health gradient have largely focused on the attainment of education, defined as the number of years of education or the academic degree obtained. This marker has limitations, however. It is limited in scope, emphasizing the quantity of education without regard to other features of education such as quality. It is also retrospective, typically calculated in adulthood after formal education has ceased. Finally, it provides little insight into the processes underlying or promoted by education that may be driving connections to adult health. In this context, there is a critical need to build on this work from a developmental perspective. It is necessary to consider educational experiences more broadly and to consider education in the period of childhood when critical pathways to adult health are likely formed. The delineation of these pathways will help pinpoint the health promoting features of education early in life, offering new opportunities for intervention in childhood to maintain health long term.

In the current study, we leveraged data from the landmark Eunice Kennedy Shriver National Institute of Child Health and Human Development (NICHD) Study of Early Child Care and Youth Development (SECCYD) and the 30-year follow-up, the Study of Health in Early and Adult Life (SHINE), to examine potential mediators of life course associations between early education and adult cardiometabolic health. Specifically, mediation was tested across a breadth of educational experiences in the period of elementary school, including student social competence and academic achievement, student-teacher relationship quality, and features of the classroom environment associated with a composite of metabolic syndrome in adulthood. The mediators of interest focused on adult SES, including adult income and educational level, and adult health behaviors, including adult diet quality, activity level, sleep duration, and smoking status. The current study is uniquely positioned to examine the education-health gradient in this novel developmental framework, which is expected to reveal new information about the origins of this link.

## Methods

### Participants

Participants were in the NICHD SECCYD and 30-year follow-up, SHINE. The NICHD SECCYD (1991-2009) was a prospective multisite study of children and their families focused on child health and development between birth and age 15.5 years.^31^ The follow-up, SHINE (2018-2022), entailed a single study visit to obtain social, behavioral, and health information in adulthood (aged 26-31 years).^32^ Details for both studies^31,32^ and study flow diagram appear in the eFigure in Supplement 1. The Strengthening the Reporting of Observational Studies in Epidemiology (STROBE) reporting guideline for cohort studies was followed in the current report. NICHD SECCYD informed consent and assent were obtained from parents and children, respectively, and the study was approved by the institutional review board of each university-based site. SHINE informed consent was obtained from the participants, and the study was approved by the institutional review board of the University of Washington.

The NICHD SECCYD took place at 10 geographically diverse sites in the US. All mother-infant dyads of infants born in preselected 24-hour intervals at participating hospitals were screened. Exclusions were mother younger than 18 years; non-English speaking; serious medical problem (mother or infant); infant placed for adoption; and refusal to complete screening. A total of 8986 mother-infant dyads were screened with 3015 families deemed eligible. In alignment with recruitment goals (eg, at least 10% single-parent households), 1526 families were recontacted for participation. Of these, 1364 families were enrolled.

Following completion of the NICHD SECCYD, 946 adolescent participants and their parents agreed to be recontacted for future studies. At the time of SHINE, 927 participants remained in the sample due to 14 who rescinded their consent and 5 who died. SHINE recruitment focused on the same sites, but augmented efforts to reach participants who had moved. Temporary exclusions were current pregnancy or breastfeeding and current cold or flu symptoms. The final SHINE sample included 705 of 927 participants (76.1%). The current study included 700 participants. Five participants who no longer identified as their sex assigned at birth were excluded due to the potential impact of gender-affirming treatments on the health outcomes of interest.

Attrition analyses were performed to examine the sociodemographic characteristics, including biological sex, race and ethnicity, and parental SES, of participants in SHINE vs individuals in the NICHD SECCYD who did not participate in SHINE. Logistic regression models showed the odds of retention in SHINE were higher in women (odds ratio [OR], 1.55; 95%, CI, 1.247-1.920) and in individuals with parents of higher SES (OR, 1.14; 95% CI, 1.061-1.213).

### Measures

#### Covariates

Covariates included biological sex (female vs male), race and ethnicity, child body mass index (BMI) percentile, and parental SES. The race and ethnicity categories were Black, Latino, White, and other, which combined Asian or Pacific Islander, American Indian or Alaskan Native, and multiple race due to their small numbers. The race and ethnicity categories were defined by the investigators at the beginning of the NICHD SECCYD using US Census classifications, and the mothers reported on the race and ethnicity of their children. Child BMI percentile was calculated as the mean of BMI percentile values at child ages 24, 36, and 54 months using the 2000 CDC BMI-for-age clinical growth charts. Parental SES was calculated as a unit-standardized composite of mean parental education (computed from maternal and paternal educational attainment at child ages 1 month) and mean family income-to-needs ratio (computed from income-to-needs ratio values at child ages 1, 6, 15, 24, 36, and 54 months).

#### Educational Indicators in Childhood

Educational indicators included student social competence, student-teacher relationship quality, classroom emotional quality, classroom instructional quality, and academic achievement. Aggregates of these measures were taken to represent the integrated educational experiences of students over time with higher scores reflecting more favorable experiences.

Student social competence was assessed by teacher reports each year between kindergarten and grade 6 using the Social Skills Rating System—Teacher form,^33^ rating skills in areas of student cooperation, assertion, and self-control. Standard scores were calculated for each grade (α = .90 to 0.94) and the mean of the 7 grade-level values was then taken to form an overall student social competence score (α = .82).

Student-teacher relationship quality was assessed by teacher reports each year between kindergarten and grade 6 using the Student-Teacher Relationship Scale,^34^ characterizing the degree of conflict and closeness in the relationship. The mean of items was taken for each grade level (α = .86 to 0.89) and the mean of the 7 grade-level values was then taken to form an overall student-teacher relationship quality score (α = .78).

Classroom emotional quality and classroom instructional quality were assessed in grades 1, 3, and 5 using the Classroom Observation System.^35,36^ For classroom emotional quality, trained observers used a scale between 1 (uncharacteristic) and 7 (extremely characteristic) to rate classroom positive emotional climate, negative emotional climate, and overcontrol. The mean was taken for each grade (α = .57 to 0.83) and the mean of the 3 grade-level values was then taken to form an overall classroom emotional quality score. For classroom instructional quality, the same scale was used to rate content that varied by grade, focusing on classroom management, evaluative feedback, instructional methods, and use of time. The mean was taken for each grade (α = .53 to 0.75) and the mean of the 3 grade-level values was then taken to form an overall classroom instructional quality score.

Academic achievement was assessed using age-appropriate reading and math subtests from the Woodcock-Johnson Psycho-Education Battery-Revised,^37^ administered at child age 54 months, grades 1, 3, 5, and child age 15 years. Standard scores were calculated for each subtest and the mean was taken for each age or grade level (α = .73 to 0.89). The mean of the 5 age and grade-level values was then taken to form an overall academic achievement score (α = .92).

#### Adult Candidate Mediators

Candidate mediators were adult income and educational attainment and adult health behaviors, including diet quality, activity level, sleep duration, and smoking status. Income was assessed by self-report of total household income divided by the total number of individuals dependent on the income. Educational attainment was assessed by self-report in categories ranging from 1 (no high school diploma) to 9 (doctoral degree). Diet quality was assessed by conducting three 24-hour diet recall interviews using the Automated Self-Administered 24-Hour Dietary Assessment,^38^ which were then scored using the Healthy Eating Index-2015^39^ scoring system and the mean across interviews taken. Activity level and sleep duration were assessed over a period of 7 consecutive days using a single activity monitor worn on the right hip during the day for activity assessment and on the wrist of the nondominant hand during the night for sleep assessment. Data were scored using software (ActiLife; Actigraph, LLC), calculating the total minutes of moderate to vigorous activity per day from which the mean over days was taken as well as total hours of sleep per night from which the mean over nights was taken. Smoking status was assessed by self-report coded dichotomously in categories of 0 (never smoking) vs 1 (current/past smoking).

#### Adult Cardiometabolic Risk

An index of metabolic syndrome was created by combining cardiometabolic risk (CMR) indicators, including waist circumference (WC), systolic blood pressure (SBP), diastolic blood pressure (DBP), levels of hemoglobin A1c (HbA_1c_), C-reactive protein (CRP), and high-density lipoprotein (HDL). After reverse scoring HDL, all 6 indicators were standardized, summed, and restandardized to form a single composite of adult CMR.

WC was assessed using a tension-controlled tape measure positioned at the participant’s midpoint between the iliac crest and lowest rib. The measurement was taken on the exhalation and repeated until consecutive measurements were within 0.2 cm, and the mean of the final two values was taken to form the WC indicator. SBP and DBP were assessed using an automated blood pressure monitor following a 5-minute rest period with the participant seated in a relaxed position and the cuff positioned on the left arm. The mean of 3 consecutive measurements was taken to form separate SBP and DBP indicators. Levels of HbA_1c_, CRP, and HDL were assessed from blood that was drawn following an overnight fast and with other timed restrictions (eg, cessation of nicotine in past hour). HbA_1c_ was assayed using an enzyme immunoassay kit (ELISA Kit, E4656, ABcam); the inter-assay coefficient of variation was 10.4%, and the intra-assay coefficient of variation was 8.1%. CRP was assayed using an immunoassay kit (ELISA Kit, KHA0031; Invitrogen/Thermo Fisher Scientific); the interassay coefficient of variation was 9.9%, and the intra-assay coefficient of variation was 6.1%. HDL was assayed using conventional enzymatic methods.

### Statistical Analysis

Multiple imputation with estimated mean matching was performed for missing data using SPSS Statistics version 28.0.1.0 (IBM Corporation). Models were estimated in 10 imputed datasets and the results pooled. The sample included 700 observations. All variables had at least 75% of responses observed. Details regarding missing data appear in eTable 1 in Supplement 1. Analyses included a series of linear equations with the CMR composite regressed onto the candidate mediator as well as the child educational indicators and covariates, plus the candidate mediator regressed onto the child educational indicators. When examining adult smoking status as a mediator, analyses included a series of linear and logistic equations in which associations between the independent variables and smoking status corresponded to a one-unit change centered around the independent variable’s mean. For the mediated associations, the percentage of the corresponding total association explained by the mediated pathways is reported for models with no negative confounding. Analyses were performed in SAS version 15.3 (SAS Institute Inc). The 95% CIs and *P* values for indirect associations were estimated via bootstrap with 1000 bootstrap samples and using the partial posterior *P* value method, respectively.

## Results

### Descriptives

The sample included 700 participants (74 female [53.4%]; mean [SD] age, 28.6 [1.2] years; 70 [10.0%] Black, 45 [6.4%] Latino, 552 [78.9%] White, and 33 [4.7%] other) (Table 1). Regarding SES, in childhood, 50.7% of participants had at least one parent with a college degree or higher. In adulthood, 55.7% of participants had a college degree or higher. Regarding health status, in childhood, 21.6% had overweight or obesity at 54 months and, in adulthood, 53.6% exceeded the risk threshold for WC. Other adult indicators showed 30.0% had hypertension and 34.2% had prediabetes or diabetes. Bivariate correlations among the primary variables are provided in eTable 2 in Supplement 1.

**Table 1.** Sample Description Information^a^

Variable	Participants, No. (%)
Sociodemographics	
Age (at adult assessment), mean (SE), y	28.6 (1.20)
Biological sex	
Female	374 (53.4)
Male	326 (46.6)
Race and ethnicity	
Black	70 (10.0)
Latino	45 (6.4)
White	552 (78.9)
Other^b^	33 (4.7)
Child BMI percentile	
BMI percentile, mean (SE), 24, 36, 54 mo	56.8 (0.90)
Overweight or obesity (24 mo)	102.9 (14.7)
Overweight or obesity (36 mo)	113.0 (16.2)
Overweight or obesity (54 mo)	151.3 (21.6)
Parental SES	
Mother or father, ≥college degree	355 (50.7)
Family income-to-needs ratio, mean (SE), 1, 6, 15, 36, 54 mo	3.6 (.09)
Families in poverty (based on mean income-to-needs ratio)	55.1 (7.9)
Early educational indicators, mean (SE)	
Student social competence (teacher reported)	104.1 (0.39)
Student-teacher relationship (teacher reported)	64.2 (0.22)
Classroom emotional quality (observer rated)	5.4 (0.02)
Classroom instructional quality (observer rated)	4.2 (0.02)
Academic achievement (math/reading assessment)	108.8 (0.42)
Adult cardiometabolic risk indicators	
WC, mean (SE), cm	92.2 (0.68)
WC risk (≥80 cm women, ≥94 cm men)	375.2 (53.6)
SBP, mean (SE), mm Hg	115.1 (0.51)
DBP, mean (SE), mm Hg	73.3 (0.41)
BP risk (SBP ≥130 or DBP ≥80)	210.0 (30.0)
HbA_1c_	5.4 (0.12)
HbA_1c_ risk (≥5.7%)	239.4 (34.2)
CRP, mean (SE), mg/L	4.6 (0.20)
CRP risk, mean (SE), ≥10 mg/L	87.5 (12.5)
HDL, mean (SE), mg/dL	54.2 (0.66)
HDL risk (<50 mg/dL women, <40 mg/dL men)	170.8 (24.4)
Adult mediators	
Adult annual household income, mean (SE), $	82 202 (2359)
Adult educational attainment (≥college degree)	389.7 (55.7)
Adult diet quality, mean (SE), HEI score	50.3 (0.44)
Adult activity level, mean (SE), MVPA min	75.8 (2.30)
Adult sleep duration, mean (SE), h	7.3 (0.04)
Adult smoking status (current/past smoking)	192.5 (27.5)

Abbreviations: BMI, body mass index; BP, blood pressure; CRP, C-reactive protein; DBP, diastolic blood pressure; HbA_1c_, hemoglobin A1c; HDL, high-density lipoprotein; HEI, health eating index; MVPA, moderate to vigorous physical activity; SBP, systolic blood pressure; SES, socioeconomic status; WC, waist circumference.

SI conversion factors: To convert CRP to milligrams per liter, multiply by 10; HbA_1c_ to proportion of total hemoglobin, multiply by 0.01; HDL to millimoles per liter, multiply by 0.0259.

^a^ Mean (SE), or No. (%) are reported from multiply imputed data for 700 participants.

^b^ Other race and ethnicity included Asian or Pacific Islander (n = 8), American Indian or Alaska Native (n = 1), multiple race and ethnicity (n = 24).

### Mediation Analyses

Mediation analyses revealed adult income and adult diet quality significantly mediated associations between child educational indicators and adult CMR, controlling for covariates. These results are described for each child educational indicator. Additional results are located in eTables 3-8 in Supplement 1, presenting details about the covariates as well as models for which mediation was nonsignificant (educational attainment, activity level, sleep duration, and smoking status).

#### Adult Income as a Mediator

##### Student social competence

**Figure 1.**
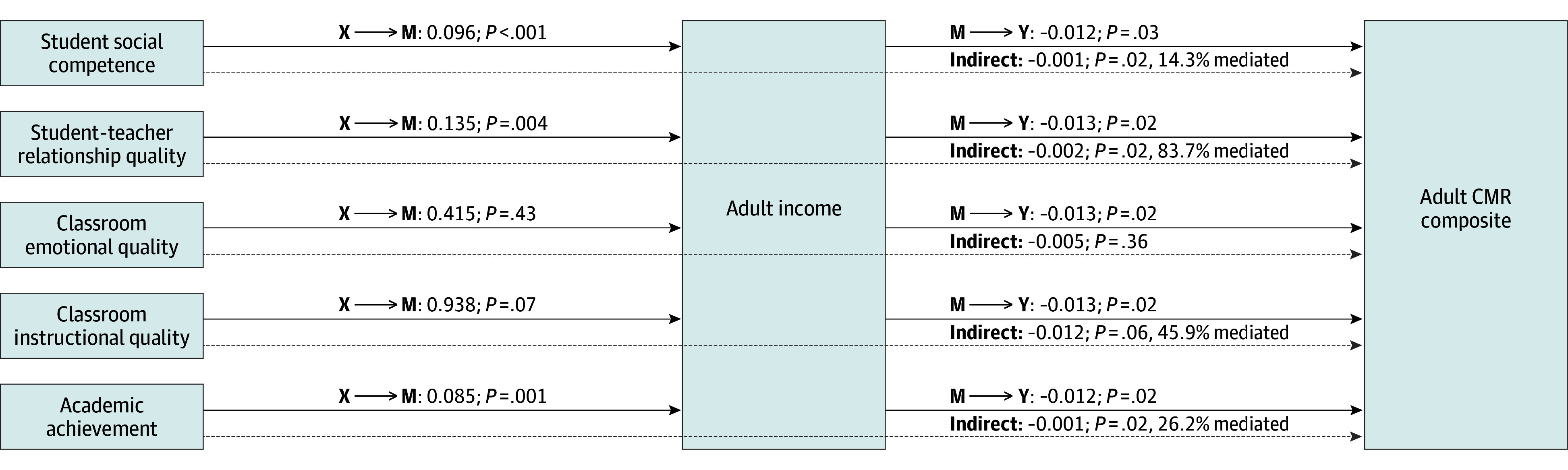
Adult Income as a Mediator Between Educational Indicators in Childhood and the Adult Cardiometabolic Risk (CMR) Composite Results are reported from separate mediation models adjusted for covariates (biological sex, race and ethnicity, parental socioeconomic status [SES], and child body mass index [BMI] percentile). The solid arrows represent direct paths between the educational indicators in childhood (X) and adult income (M) and the direct paths between adult income (M) and the adult CMR composite (Y). The dotted arrows represent the indirect (mediated) paths, showing adult income mediated effects of student social competence, student-teacher relationship quality, classroom instructional quality, and academic achievement on the adult CMR composite.

In model 1a, higher student social competence was associated with a higher adult income (path a: *b* = 0.0964; 95% CI, 0.044-0.149; *P* < .001) and higher adult income was associated with lower adult CMR (path b: *b* = −0.0117; 95% CI, −0.022 to −0.001; *P* = .03). The indirect effect showed adult income significantly mediated the association between higher student social competence and lower adult CMR (*b* = −0.0011; 95% CI, −0.002 to −0.0003; *P* = .02), with 14.3% of the association between student social competence and adult CMR mediated by adult income (Table 2, Figure 1).

**Table 2.** Mediation Between the Child Educational Indicators and Adult CMR by Adult Income, Adjusted for Covariates^a^

Mediation model	Estimate, *b* (95% CI)	*P* value
1A: **Student social competence (X) → adult income (M) → CMR (Y)**		
X → Y (total effect, path c)^b^	−0.0079 (−0.0155 to −0.0004)	.04
X → M (path a)^c^	0.0964 (0.0435 to 0.1494)	<.001
M → Y (path b)^d^	−0.0117 (−0.0224 to −0.0010)	.03
X → Y (direct effect, path c’)^d^	−0.0068 (−0.0144 to 0.0007)	.08
X → M → Y (indirect effect)^e^	−0.0011 (−0.0024 to −0.0003)	.02
**1B: Student-teacher relationship quality (X) → adult income (M) → CMR (Y)**		
X → Y (total effect, path c)^b^	−0.0021 (−0.0146 to 0.0106)	.75
X → M (path a)^c^	0.1352 (0.0422 to 0.2282)	.004
M → Y (path b)^d^	−0.0129 (−0.0236 to −0.0023)	.02
X → Y (direct effect, path c’)^d^	−0.0003 (−0.0127 to 0.0126)	.96
X → M → Y (indirect effect)^e^	−0.0018 (−0.0037 to −0.0005)	.02
**1C: Classroom emotional quality (X) → adult income (M) → CMR (Y)**		
X → Y (total effect, path c)^b^	−0.0051 (−0.1150 to 0.1155)	.94
X → M (path a)^c^	0.4153 (−0.6173 to 1.4479)	.43
M → Y (path b)^d^	−0.0130 (−0.0236 to −0.0024)	.02
X → Y (direct effect, path c’)^d^	0.0004 (−0.1089 to 0.1212)	.99
X → M → Y (indirect effect)^e^	−0.0054 (−0.0203 to 0.0067)	.36
**1D: Classroom instructional quality (X) → adult income (M) → CMR (Y)**		
X → Y (total effect, path c)^b^	−0.0264 (−0.1642 to 0.1006)	.72
X → M (path a)^c^	0.9381 (−0.0816 to 1.9578)	.07
M → Y (path b)^d^	−0.0129 (−0.0235 to −0.0023)	.02
X → Y (direct effect, path c’)^d^	−0.0143 (−0.1503 to 0.1114)	.84
X → M → Y (indirect effect)^e^	−0.0121 (−0.0339 to −0.0013)	.06
**1E: Academic achievement (X) → adult income (M) → CMR (Y)**		
X → Y (total effect, path c)^b^	−0.0040 (−0.0115 to 0.0025)	.29
X → M (path a)^c^	0.0851 (0.0339 to 0.1363)	.001
M → Y (path b)^d^	−0.0124 (−0.0231 to −0.0018)	.02
X → Y (direct effect, path c’)^d^	−0.0030 (−0.0104 to 0.0038)	.43
X → M → Y (indirect effect)^e^	−0.0011 (−0.0024 to −0.0004)	.02

Abbreviation: CMR, cardiometabolic risk.

^a^ Covariates included biological sex, race and ethnicity, child body mass index percentile, and parental socioeconomic status. The arrow symbol (→) denotes a path between the variables.

^b^ Y is regressed onto X and covariates, excluding M.

^c^ M is regressed onto X and covariates.

^d^ Y is regressed onto X, M, and covariates.

^e^ Mediated effects of X on Y via M.

##### Student-teacher relationship quality

In model 1b, higher student-teacher relationship quality was associated with higher adult income (path a: *b* = 0.1352; 95% CI, 0.042-0.228; *P* = .004) and higher adult income was associated with lower adult CMR (path b: *b* = −0.0129; 95% CI, 0.042-0.228; *P* = .02). The indirect effect showed adult income significantly mediated the association between higher student-teacher relationship quality and lower adult CMR (*b* = −0.0018; 95% CI, −0.004 to −0.001; *P* = .02), with 83.7% of the association between student-teacher relationship quality and adult CMR mediated by adult income.

##### Classroom emotional quality

In model 1c, classroom emotional quality was not associated with adult income (path a: *b* = 0.4153; 95% CI, −0.617 to 1.448; *P* = .43), but higher adult income was associated with lower adult CMR (path b: *b* = −0.0130; 95% CI, −0.024 to −0.002; *P* = .02). The indirect effect showed adult income did not mediate the association between classroom emotional quality and adult CMR (*b* = −0.005; 95% CI, −0.020 to 0.007; *P* = .36).

##### Classroom instructional quality

In model 1d, higher classroom instructional quality was not associated with adult income (path a: *b* = 0.9381; 95% CI, −0.082 to 1.958; *P* = .07) and higher adult income was associated with lower adult CMR (path b: *b* = −0.0129; 95% CI, −0.024 to −0.002; *P* = .02). The indirect effect showed adult income mediated the association between higher classroom instructional quality and lower adult CMR (*b* = −0.0121; 95% CI, −0.034 to −0.001; *P* = .06 for trend), with 45.9% of the association between classroom instructional quality and adult CMR mediated by adult income.

##### Academic achievement

In model 1e, higher academic achievement was associated with higher adult income (path a: *b* = 0.0850; 95% CI, 0.034-0.136; *P* = .001) and higher adult income was associated with lower adult CMR (path b: *b* = −0.0124; 95% CI, −0.023 to −0.002; *P* = .02). The indirect effect showed adult income significantly mediated the association between higher academic achievement and lower adult CMR (*b* = −0.0011; 95% CI, −0.002 to −0.0004; *P* = .02), with 26.2% of the association between academic achievement and adult CMR mediated by adult income.

#### Adult Diet Quality as a Mediator

##### Student social competence

**Figure 2.**
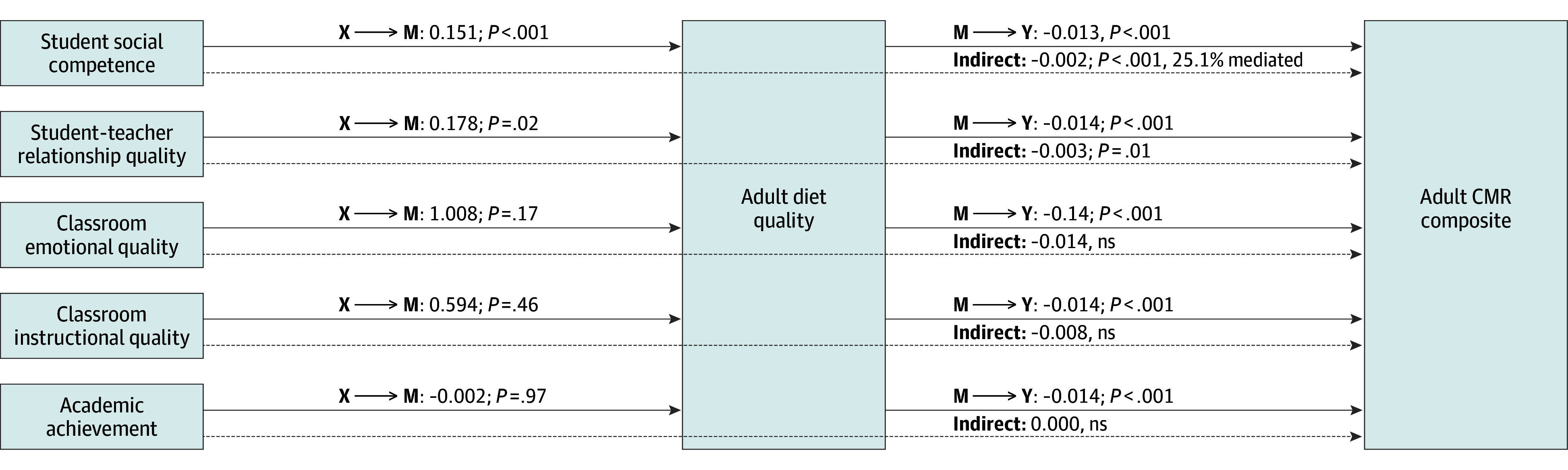
Adult Diet as a Mediator Between Educational Indicators in Childhood and the Adult Cardiometabolic Risk (CMR) Composite Results are reported from separate mediation models adjusted for covariates (biological sex, race and ethnicity, parental socioeconomic status [SES], and child body mass index [BMI] percentile). The solid arrows represent direct paths between the educational indicators in childhood (X) and adult diet quality (M) and the direct paths between adult diet quality (M) and the adult CMR composite (Y). The dotted arrows represent the indirect (mediated) paths, showing adult diet quality mediated effects of student social competence and student-teacher relationship quality on the adult CMR composite.

In model 2a, higher student social competence was associated with higher adult diet quality (path a: *b* = 0.1512; 95% CI, 0.067-0.235; *P* < .001) and higher adult diet quality was associated with lower adult CMR (path b: *b* = −0.0132; 95% CI, (−0.020 to −0.006; *P* < .001). The indirect effect showed adult diet quality significantly mediated the association between higher student social competence and lower adult CMR (*b* = −0.0020; 95% CI, −0.004 to −0.001; *P* = .000), with 25.1% of the association between student social competence and adult CMR mediated by adult diet quality (Table 3, Figure 2).

**Table 3.** Mediation Between the Child Educational Indicators and Adult CMR by Adult Diet Quality, Adjusted for Covariates^a^

Mediation model	Estimate (95% CI)	*P* value
**2A: Student social competence (X) → adult diet quality (M) → CMR (Y)**		
X → Y (total effect, path c)^b^	−0.0079 (−0.0153 to −0.0003)	.04
X → M (path a)^c^	0.1512 (0.0674 to 0.2349)	<.001
M → Y (path b)^d^	−0.0132 (−0.0203 to −0.0060)	<.001
X → Y (direct effect, path c’)^d^	−0.0059 (−0.0135 to 0.0016)	.13
X → M → Y (indirect effect)^e^	−0.0020 (−0.0040 to −0.0008)	<.001
**2B: Student-teacher relationship quality (X) → adult diet quality (M) → CMR (Y)**		
X → Y (total effect, path c)^b^	−0.0021 (−0.0162 to 0.0112)	.75
X → M (path a)^c^	0.1778 (0.0308 to 0.3247)	.02
M → Y (path b)^d^	−0.0139 (−0.0210 to −0.0068)	<.001
X → Y (direct effect, path c’)^d^	0.0004 (−0.0137 to 0.0135)	.96
X → M → Y (indirect effect)^e^	−0.0025 (−0.0052 to −0.0006)	.01
**2C: Classroom emotional quality (X) → adult diet quality (M) → CMR (Y)**		
X → Y (total effect, path c)^b^	−0.0051 (−0.1251 to 0.1141)	.94
X → M (path a)^c^	1.0081 (−0.4347 to 2.4509)	.17
M → Y (path b)^d^	−0.0139 (−0.0210 to −0.0068)	<.001
X → Y (direct effect, path c’)^d^	0.0088 (−0.1104 to 0.1278)	.89
X → M → Y (indirect effect)^e^	−0.0139 (−0.0391 to 0.0053)	.14
**2D: Classroom instructional quality (X) → adult diet quality (M) → CMR (Y)**		
X → Y (total effect, path c)^b^	−0.0264 (−0.1506 to 0.1031)	.72
X → M (path a)^c^	0.5937 (−0.9834 to 2.1708)	.46
M → Y (path b)^d^	−0.0139 (−0.0209 to −0.0068)	<.001
X → Y (direct effect, path c’)^d^	−0.0181 (−0.1424 to 0.1103)	.80
X → M → Y (indirect effect)^e^	−0.0082 (−0.0360 to 0.0118)	.42
**2E: Academic achievement (X) → adult diet quality (M) → CMR (Y)**		
X → Y (total effect, path c)^b^	−0.0040 (−0.0112 to 0.0029)	.29
X → M (path a)^c^	−0.0018 (−0.0853 to 0.0816)	.97
M → Y (path b)^d^	−0.0139 (−0.0210 to −0.0068)	<.001
X → Y (direct effect, path c’)^d^	−0.0041 (−0.0113 to 0.0030)	.29
X → M → Y (indirect effect)^e^	0.0000 (−0.0010 to 0.0012)	.96

^a^ Covariates included biological sex, race and ethnicity, child body mass index percentile, and parental socioeconomic status. The arrow symbol (→) denotes a path between the variables.

^b^ Y is regressed onto X and covariates, excluding M.

^c^ M is regressed onto X and covariates.

^d^ Y is regressed onto X, M, and covariates.

^e^ Mediated effects of X on Y via M.

##### Student-teacher relationship quality

In model 2b, higher student-teacher relationship quality was associated with higher adult diet quality (path a: *b* = 0.1778; 95% CI, 0.031 to 0.325; *P* = .02) and higher adult diet quality was significantly related to lower adult CMR (path b: *b* = −0.0139; 95% CI, −0.021 to −0.007; *P* < .001). The indirect effect showed adult diet quality significantly mediated the association between higher student-teacher relationship quality and lower adult CMR (*b* = −0.0025; 95% CI, −0.005 to −0.001; *P* = .01).

##### Classroom emotional quality, classroom instructional quality, and academic achievement

In models 2c-2e, the child educational exposure was not associated with adult diet quality, but higher adult diet quality was associated with lower adult CM (path b) for classroom emotional quality (*b* = −0.013; 95% CI, −0.024 to −0.002; *P* = .02), classroom instructional quality (*b* = −0.013; 95% CI, −0.024 to −0.002; *P* = .02), and academic achievement (*b* = −0.012; 95% CI, −0.023 to −0.002; *P* = .02). The indirect effects were nonsignificant.

## Discussion

The role of SES as a social determinant of health is now well established. However, the developmental pathways linking SES to health remain poorly understood. Using longitudinal data from the landmark SECCYD and 30-year follow-up, SHINE, findings in the current study revealed that favorable educational experiences in elementary school were associated with better cardiometabolic health in adulthood, partially through having a higher income in adulthood as well as through having healthier dietary habits in adulthood. These associations were observed even with adjustment for key covariates measured prior to the start of elementary school, including parental SES and childhood health status.

In the current study, early educational experiences were examined broadly, considering a range of student, student-teacher, and classroom indicators. For 4 of 5 of these indicators, including student social competence, student-teacher relationship quality, classroom instructional quality, and academic achievement, adult income emerged as a mediator between these exposures and adult CMR. Although speculative, positive educational experiences may impact adult CMR through skillsets that support the attainment of a higher income and through the subsequent benefits of having a higher income on cardiometabolic health. The protective effects of income are likely due to the availability of monetary resources for health-promoting activities and for health care services.^40,41,42^ With respect to links between specific educational indicators and adult income, it is possible that classroom instructional quality and academic achievement may have direct impacts on opportunities for career advancement and higher wages.^43,44,45^ Other constructs, such as student social competence and student-teacher relationship quality, may operate through self-regulatory and interpersonal skills, which have been shown to predict positive academic, psychosocial, and health outcomes.^46,47,48,49,50,51,52,53,54^ Together, findings are notable in suggesting both early academic and social experiences, which themselves may be partially shaped by early child care environments,^55^ may embed protections for cardiometabolic health in adulthood via adult income.

Findings are also notable in revealing that adult educational attainment did not mediate associations between early educational experiences and adult CMR. This was unexpected as most studies^17,18,19,20,21,22,23^ have focused on the significance of educational attainment in association with adult health. This highlights that while educational attainment may predict adult health, it may not be a pathway through which early educational experiences influence health. This distinction may mean that factors such as higher income may play a practical role in helping an individual to acquire resources that are needed for better health, but correlated factors such as educational attainment do not. This finding may be especially salient in the US health care system (vs one based on universal health care) as evidence shows monetary resources are needed to navigate the US system.^56,57,58^ In this way, it may be the quality of early educational experiences that impacts pathways to health, irrespective of the quantity of education.

In parallel, adult diet quality also emerged as a mediator between early educational experiences, including student social competence and student-teacher relationship quality, and adult CMR. Although speculative, positive educational experiences may impact adult CMR through skillsets that support the adoption of a high-quality diet and through the subsequent impact of having a high-quality diet on the protection of cardiometabolic health. The protective effects of diet are likely due to the well-known benefits of healthy eating habits.^59^ Notably, mediation was observed for student social competence and student-teacher relationship quality only, suggesting self-regulatory and interpersonal skills impact healthy eating^60,61^ but not academic skills. Moreover, findings in the current study revealed that the other examined health behaviors, including adult activity level, adult sleep duration, and adult smoking, did not mediate associations between early educational experiences and adult CMR. This highlights the role of adult diet quality uniquely. Although it is common to focus on diet as a way to improve health, such interventions have generally had modest results.^62,63^ It is notable, in contrast, that findings in the current study suggest early educational experiences may embed healthy eating habits long term, a potentially enduring path to better cardiometabolic health.

### Strengths and Limitations

Strengths of the current study were the use of longitudinal data from the landmark SECCYD and 30-year follow-up, SHINE, offering state-of-the-art assessments in the key areas of interest. Early educational indicators were obtained repeatedly over time and from multiple sources, including students, teachers, trained observers, and established testing tools. The CMR composite, indexing metabolic syndrome, was based on best practices for assessments in areas of central adiposity, blood pressure, insulin resistance, inflammation, and dyslipidemia. Limitations of the current study were the small number of participants from racial and ethnic minority backgrounds, limiting generalizability of the results. Additionally, attrition analyses showed women and individuals from higher SES backgrounds as children were more likely to participate in SHINE. This raises the likelihood of bias in the current sample toward greater socioeconomic advantage and reduced representation of the original SECCYD. Another weakness is that only one assessment was performed in adulthood, limiting the examination of the adult health behaviors and the CMR composite over time. Finally, it is important to be cautious in the interpretation of the current findings as the current study was observational and cannot address whether associations between the constructs of interest were causal in nature. Likewise, it is important to highlight that the effect sizes for the educational indicators and mediators were quite small and notably smaller in comparison with key covariates, such as parental SES.

## Conclusions

The results of this cohort study suggest that the long-term health benefits of early educational experiences may be rooted in the attainment of skills in both academic and nonacademic areas and partially operate through mechanisms, including adult income and adult diet quality. The implications of this work are that skill sets attained in school settings might be augmented to protect cardiometabolic health over the life course. Such intervention efforts should focus on skills in areas of academics as well as socioemotional health and be considered for targeting outside traditional school settings.

## Notes

Supplement 1. **eFigure.** Flow Diagram of Screening and Eligibility Criteria Used in the NICHD SECCYD and SHINE **eTable 1.** Number of Observations for Each of the Primary Variables **eTable 2.** Bivariate Correlations Among the Primary Variables **eTable 3.** Mediated Effects of the Child Educational Indicators on Adult CMR via Adult Income, Adjusted for Covariates **eTable 4.** Mediated Effects of the Child Educational Indicators on Adult CMR via Adult Diet Quality, Adjusted for Covariates **eTable 5.** Mediated Effects of the Child Educational Indicators on Adult CMR via Adult Educational Attainment, Adjusted for Covariates **eTable 6.** Mediated Effects of the Child Educational Indicators on Adult CMR via Adult Activity Level, Adjusted for Covariates **eTable 7.** Mediated Effects of the Child Educational Indicators on Adult CMR via Adult Sleep Duration, Adjusted for Covariates **eTable 8.** Mediated Effects of the Child Educational Indicators on Adult CMR via Adult Smoking Status, Adjusted for Covariates

Supplement 2.
Data Sharing Statement
