# Supplementary material for: Mediators of the Association Between Child Education and Adult Health
Source: JAMA Netw Open. 2025 May 23;8(5):e258855. doi: 10.1001/jamanetworkopen.2025.8855 (PMC12102707; doi:10.1001/jamanetworkopen.2025.8855)
Supplement: Supplement 1. — eFigure. Flow Diagram of Screening and Eligibility Criteria Used in the NICHD SECCYD and SHINE eTable 1. Number of Observations for Each of the Primary Variables eTable 2. Bivariate Correlations Among the Primary Variables eTable 3. Mediated Effects of the Child Educational Indicators on Adult CMR via Adult Income, Adjusted for Covariates eTable 4. Mediated Effects of the Child Educational Indicators on Adult CMR via Adult Diet Quality, Adjusted for Covariates eTable 5. Mediated Effects of the Child Educational Indicators on Adult CMR via Adult Educational Attainment, Adjusted for Covariates eTable 6. Mediated Effects of the Child Educational Indicators on Adult CMR via Adult Activity Level, Adjusted for Covariates eTable 7. Mediated Effects of the Child Educational Indicators on Adult CMR via Adult Sleep Duration, Adjusted for Covariates eTable 8. Mediated Effects of the Child Educational Indicators on Adult CMR via Adult Smoking Status, Adjusted for Covariates [file jamanetwopen-e258855-s001.pdf]

Supplementary Online Content

Bleil ME, Roisman GI, Hamilton DT, et al. Mediators of the association between child education and adult health. *JAMA Netw Open.* 2025;8(5):e258855 doi:10.1001/jamanetworkopen.2025.8855

eFigure. Flow Diagram of Screening and Eligibility Criteria Used in the NICHD SECCYD and SHINE

eTable 1. Number of Observations for Each of the Primary Variables

eTable 2. Bivariate Correlations Among the Primary Variables

eTable 3. Mediated Effects of the Child Educational Indicators on Adult CMR via Adult Income, Adjusted for Covariates

eTable 4. Mediated Effects of the Child Educational Indicators on Adult CMR via Adult Diet Quality, Adjusted for Covariates

eTable 5. Mediated Effects of the Child Educational Indicators on Adult CMR via Adult Educational Attainment, Adjusted for Covariates

eTable 6. Mediated Effects of the Child Educational Indicators on Adult CMR via Adult Activity Level, Adjusted for Covariates

eTable 7. Mediated Effects of the Child Educational Indicators on Adult CMR via Adult Sleep Duration, Adjusted for Covariates

eTable 8. Mediated Effects of the Child Educational Indicators on Adult CMR via Adult Smoking Status, Adjusted for Covariates

This supplementary material has been provided by the authors to give readers additional information about their work.

© 2025 Bleil ME et al. *JAMA Network Open*.

eFigure. Flow Diagram of Screening and Eligibility Criteria Used in the NICHD SECCYD and SHINE

© 2025 Bleil ME et al. *JAMA Network Open*.

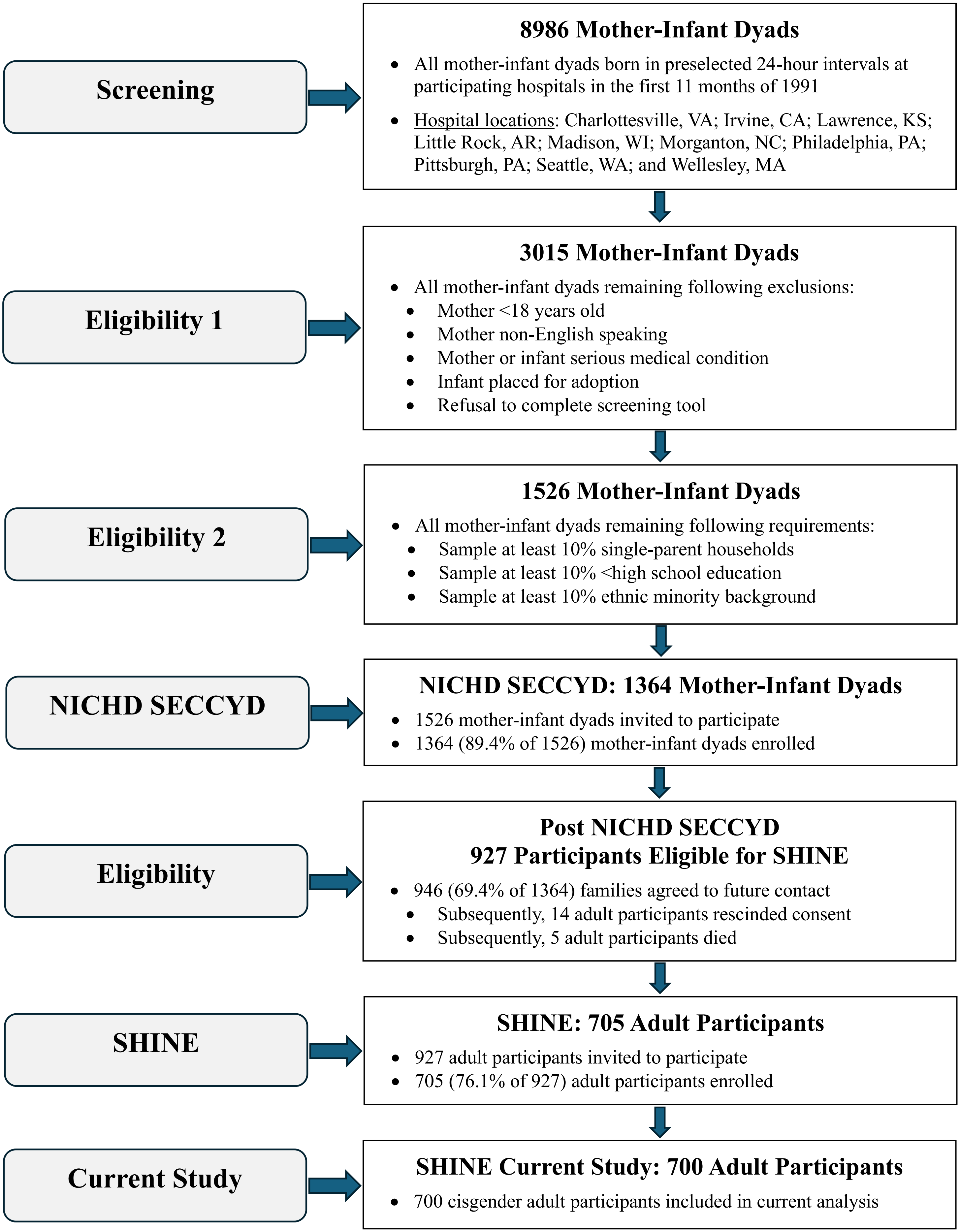

© 2025 Bleil ME et al. *JAMA Network Open*.

eTable 1. Number of Observations for Each of the Primary Variables

	**Number of observations**	**% Complete data**
**Socio-demographics:**		
Biological sex	n=700	100%
Race/ethnicity	n=700	100%
**Child BMI percentile:**		
BMI percentile (mean: 24, 36, 54 months)	n=675	96.4%
**Parental SES:**		
Parental education (mean: mother/father education level)	n=700	100%
Family income-to-needs ratio (mean: 1, 6, 15, 36, 54 months)	n=699	99.9%
**Early educational indicators:**		
Student social competence (teacher reported)	n=694	99.1%
Student-teacher relationship (teacher reported)	n=698	99.7%
Classroom emotional quality (observer rated)	n=683	97.6%
Classroom instructional quality (observer rated)	n=683	97.6%
Academic achievement (math/reading assessment)	n=699	99.9%
**Adult cardiometabolic risk indicators:**		
WC (cm)	n=646	92.3%
SBP (mm Hg)	n=644	92.0%
DBP (mm Hg)	n=644	92.0%
HbA1c	n=524	74.9%
CRP (mg/L)	n=524	74.9%
HDL (mg/dL)	n=525	75.0%
**Adult mediators:**		
Adult household income ($50,000/year+)	n=690	98.6%
Adult educational attainment (college degree+)	n=695	99.3%
Adult diet quality (HEI score)	n=661	94.4%
Adult activity level (MVPA minutes)	n=577	82.4%
Adult sleep duration (hours)	n=579	82.7%
Adult smoking status (current/past smoking)	n=694	99.1%

BMI=body mass index; SES=socioeconomic status; WC=waist circumference; SBP=systolic blood pressure; DBP=diastolic blood pressure; HbA1c=hemoglobin A1c; CRP=C-reactive protein; HDL=high-density lipoprotein; HEI=health eating index; MVPA=moderate to vigorous physical activity.

© 2025 Bleil ME et al. *JAMA Network Open*.

eTable 2. Bivariate Correlations Among the Primary Variables

	1.	2.	3.	4.	5.	6.	7.	8.	9.	10.	11.	12.	13.	14.	15.	16.	17.	18.	19.
1. Sex	-																		
2. Black	.015	-																	
3. Latino	-.059	-	-																
4. White	-.007	-	-	-															
5. Other	.036	-	-	-	-														
6. BMI-P	-.060	.017	.003	-.047	-.006	-													
7. P-SES	.014	-.275***	-.102**	.285***	.035	-.070†	-												
8. Social	-.034	-.266***	-.046	.232***	.036	-.107**	.352***	-											
9. Relation	.282***	-.224***	-.061	.220***	.029	-.120**	.257***	.747***	-										
10. Class-E	.032	-.222***	-.031	.168***	.055	-.068†	.291***	.211***	.211***	-									
11. Class-I	.072†	-.219***	-.038	.167***	.124**	-.086*	.239***	.207***	.217***	.501***	-								
12. Achieve	-.015	-.328***	-.093*	.288***	.093*	.029	.448***	.411***	.212***	.179***	.226***	-							
13. Income	-.084*	-.145***	-.003	.135***	.048	-.018	.384***	.257***	.169***	.143**	.151***	.179***	-						
14. Educ	.098**	-.177***	-.107**	.199***	.066†	-.076†	.494***	.384***	.328***	.226***	.261***	.226***	.309***	-					
15. Diet	.106**	-.097*	-.037	.088*	.033	-.008	.340***	.231***	.191***	.150***	.114**	.151***	.230***	0.273***	-				
16. Activ	-.041	.021	-.034	-.043	-.006	.042	-.013	-.072†	-.066	.040	-.059	-.088*	-.029	-0.103*	-0.021	-			
17. Sleep	.214***	-.177***	-.024	.162***	.004	-.016	.115**	.053	.110**	.064	.056	.062	.071†	.103*	.093*	-.224***	-		
18. Smok	-.190***	-.024	-.010	.024	-.066†	.104**	-.148***	-.162***	-.172***	-.055	-.125**	-.053	-.148***	-.364***	-.162***	-.125**	-.096*	-	
19. CMR	-0.281***	0.068†	0.000	-0.045	-0.048	.203***	-.219***	-.149***	-.158***	-.084*	-.098*	-.116**	-.138***	-.157***	-.233***	.035	-.132**	.128**	–

1=biological sex (female vs. male); 2=Black (vs. all); 3=Latino (vs. all); 4=White (vs. all); 5='Other' (vs. all); 6=child BMI percentile; 7=parental SES; 8=student social competence; 9=student-teacher relationship quality; 10=classroom emotional quality; 11=classroom instructional quality; 12=academic achievement; 13=adult income; 14=adult educational attainment; 15=adult diet quality; 16=adult activity level; 17=adult sleep duration; 18=adult smoking (current/past vs. never); 19=CMR composite.

*<.05; **<.01; ***<.001; †<.10

© 2025 Bleil ME et al. *JAMA Network Open*.

eTable 3. Mediated Effects of the Child Educational Indicators on Adult CMR via Adult Income, Adjusted for Covariates

**Mediation Model 3a:** Student social competence (X) → Adult income (M) → CMR (Y):		**Estimate (95% CI)**
+	X → Y (total effect, path c)	-0.0079* (-0.0155 to -0.0004)
++	X → M (path a)	0.0964*** (0.0435 to 0.1494)
	Covariates:	
	Biological sex (female vs. male)	-1.2001* (-2.1935 to -0.2068)
	Black (vs. white)	-0.3844 (-2.1439 to 1.3751)
	Latino (vs. white)	0.8533 (-1.2072 to 2.9139)
	‘Other’ (vs. white)	-1.2636 (-3.6073 to 1.0800)
	Child BMI percentile	0.0050 (-0.0150 to 0.0250)
	Parental SES	2.4567*** (1.9132 to 3.0002)
+++	M → Y (path b)	-0.0117* (-0.0224 to -0.0010)
	X → Y (direct effect, path c’)	-0.0068† (-0.0144 to 0.0007)
	Covariates:	
	Biological sex (female vs. male)	-0.5627*** (-0.7087 to -0.4168)
	Black (vs. white)	-0.0334 (-0.2973 to 0.2306)
	Latino (vs. white)	-0.1592 (-0.4759 to 0.1575)
	‘Other’ (vs. white)	-0.1977 (-0.5523 to 0.1568)
	Child BMI percentile	0.0067*** (0.0037 to 0.0097)
	Parental SES	-0.1542*** (-0.2438 to -0.0645)
++++	X → M → Y (indirect effect)	-0.0011* (-0.0024 to -0.0003)
**Mediation Model 3b:** Student-teacher relationship quality (X) → Adult income (M) → CMR (Y):		
+	X → Y (total effect, path c)	-0.0021 (-0.0146 to 0.0106)
++	X → M (path a)	0.1352** (0.0422 to 0.2282)
	Covariates:	
	Biological sex (female vs. male)	-1.7201** (-2.7601 to -0.6800)
	Black (vs. white)	-0.5239 (-2.2871 to 1.2393)
	Latino (vs. white)	0.8469 (-1.2217 to 2.9154)
	‘Other’ (vs. white)	-1.2175 (-3.5701 to 1.1351)
	Child BMI percentile	0.0044 (-0.0157 to 0.0244)
	Parental SES	2.5908*** (2.0589 to 3.1227)
+++	M → Y (path b)	-0.0129* (-0.0236 to -0.0023)
	X → Y (direct effect, path c’)	-0.0003 (-0.0127 to 0.0126)
	Covariates:	
	Biological sex (female vs. male)	-0.5572*** (-0.7106 to -0.4039)
	Black (vs. white)	0.0075 (-0.2585 to 0.2734)
	Latino (vs. white)	-0.1484 (-0.4672 to 0.1704)
	‘Other’ (vs. white)	-0.1942 (-0.5496 to 0.1613)
	Child BMI percentile	0.0069*** (0.0040 to 0.0099)
	Parental SES	-0.1705*** (-0.2578 to -0.0832)
++++	X → M → Y (indirect effect)	-0.0018* (-0.0037 to -0.0005)
**Mediation Model 3c:** Classroom emotional quality (X) → Adult income (M) → CMR (Y):		
+	X → Y (total effect, path c)	-0.0051 (-0.1150 to 0.1155)
++	X → M (path a)	0.4153 (-0.6173 to 1.4479)
	Covariates:	
	Biological sex (female vs. male)	-1.2985* (-2.3001 to -0.2968)
	Black (vs. white)	-0.8762 (-2.6451 to 0.8926)
	Latino (vs. white)	0.7211 (-1.3565 to 2.7987)
	‘Other’ (vs. white)	-1.3740 (-3.7379 to 0.9900)
	Child BMI percentile	0.0021 (-0.0179 to 0.0222)

© 2025 Bleil ME et al. *JAMA Network Open*.

	Parental SES	2.6833*** (2.1378 to 3.2287)
+++	M → Y (path b)	-0.0130* (-0.0236 to -0.0024)
	X → Y (direct effect, path c')	0.0004 (-0.1089 to 0.1212)
	Covariates:	
	Biological sex (female vs. male)	-0.5585*** (-0.7045 to -0.4124)
	Black (vs. white)	0.0087 (-0.2548 to 0.2723)
	Latino (vs. white)	-0.1480 (-0.4662 to 0.1701)
	'Other' (vs. white)	-0.1939 (-0.5496 to 0.1619)
	Child BMI percentile	0.0069*** (0.0040 to 0.0099)
	Parental SES	-0.1708*** (-0.2611 to -0.0805)
++++	X → M → Y (indirect effect)	-0.0054 (-0.0203 to 0.0067)
**Mediation Model 3d:** Classroom instructional quality (X) → Adult income (M) → CMR (Y):		
+	X → Y (total effect, path c)	-0.0264 (-0.1642 to 0.1006)
++	X → M (path a)	0.9381† (-0.0816 to 1.9578)
	Covariates:	
	Biological sex (female vs. male)	-1.3513** (-2.3530 to -0.3497)
	Black (vs. white)	-0.7222 (-2.4850 to 1.0406)
	Latino (vs. white)	0.7632 (-1.3091 to 2.8355)
	'Other' (vs. white)	-1.4157 (-3.7759 to 0.9446)
	Child BMI percentile	0.0030 (-0.0170 to 0.0231)
	Parental SES	2.6505*** (2.1167 to 3.1842)
+++	M → Y (path b)	-0.0129* (-0.0235 to -0.0023)
	X → Y (direct effect, path c')	-0.0143 (-0.1503 to 0.1114)
	Covariates:	
	Biological sex (female vs. male)	-0.5573*** (-0.7035 to -0.4111)
	Black (vs. white)	0.0045 (-0.2587 to 0.2676)
	Latino (vs. white)	-0.1488 (-0.4661 to 0.1684)
	'Other' (vs. white)	-0.1926 (-0.5486 to 0.1634)
	Child BMI percentile	0.0069*** (0.0040 to 0.0099)
	Parental SES	-0.1696*** (-0.2581 to -0.0811)
++++	X → M → Y (indirect effect)	-0.0121† (-0.0339 to -0.0013)
**Mediation Model 3e:** Academic achievement (X) → Adult income (M) → CMR (Y):		
+	X → Y (total effect, path c)	-0.0040 (-0.0115 to 0.0025)
++	X → M (path a)	0.0851** (0.0339 to 0.1363)
	Covariates:	
	Biological sex (female vs. male)	-1.2517* (-2.2458 to -0.2576)
	Black (vs. white)	-0.2712 (-2.0548 to 1.5125)
	Latino (vs. white)	1.0003 (-1.0692 to 3.0698)
	'Other' (vs. white)	-1.3820 (-3.7296 to 0.9656)
	Child BMI percentile	-0.0005 (-0.0205 to 0.0195)
	Parental SES	2.3790*** (1.8136 to 2.9445)
+++	M → Y (path b)	-0.0124* (-0.0231 to -0.0018)
	X → Y (direct effect, path c')	-0.0030 (-0.0104 to 0.0038)
	Covariates:	
	Biological sex (female vs. male)	-0.5589*** (-0.7051 to -0.4126)
	Black (vs. white)	-0.0163 (-0.2829 to 0.2503)
	Latino (vs. white)	-0.1586 (-0.4755 to 0.1583)
	'Other' (vs. white)	-0.1918 (-0.5469 to 0.1632)
	Child BMI percentile	0.0070*** (0.0041 to 0.0099)
	Parental SES	-0.1595** (-0.2541 to -0.0650)
++++	X → M → Y (indirect effect)	-0.0011* (-0.0024 to -0.0004)

Covariates included biological sex, race/ethnicity, child BMI percentile, and parental SES.
The arrow symbol (→) denotes a path between the variables.
*<.05; **<.01; ***<.001; †<.10

© 2025 Bleil ME et al. *JAMA Network Open*.

+Y regressed onto X and covariates, excluding M (covariate effects not shown)
++M regressed onto X and covariates
+++Y regressed onto X, M, and covariates
++++Mediated effects of X on Y via M

© 2025 Bleil ME et al. *JAMA Network Open*.

eTable 4. Mediated Effects of the Child Educational Indicators on Adult CMR via Adult Diet Quality, Adjusted for Covariates

	**Mediation Model 4a:** Student social competence (X) → Adult diet quality (M) → CMR (Y):	**Estimate (95% CI)**
+	X → Y (total effect, path c)	-0.0079* (-0.0153 to -0.0003)
++	X → M (path a)	0.1512*** (0.0674 to 0.2349)
	Covariates:	
	Biological sex (female vs. male)	2.4132** (0.8595 to 3.9669)
	Black (vs. white)	0.8436 (-1.9930 to 3.6801)
	Latino (vs. white)	0.4623 (-2.7124 to 3.6370)
	'Other' (vs. white)	1.5503 (-2.0960 to 5.1966)
	Child BMI percentile	0.0140 (-0.0175 to 0.0455)
	Parental SES	3.3040*** (2.4402 to 4.1678)
+++	M → Y (path b)	-0.0132*** (-0.0203 to -0.0060)
	X → Y (direct effect, path c')	-0.0059 (-0.0135 to 0.0016)
	Covariates:	
	Biological sex (female vs. male)	-0.5170*** (-0.6616 to -0.3725)
	Black (vs. white)	-0.0177 (-0.2817 to 0.2462)
	Latino (vs. white)	-0.1630 (-0.4785 to 0.1524)
	'Other' (vs. white)	-0.1626 (-0.5135 to 0.1883)
	Child BMI percentile	0.0068*** (0.0039 to 0.0098)
	Parental SES	-0.1394** (-0.2290 to -0.0499)
++++	X → M → Y (indirect effect)	-0.0020** (-0.0040 to -0.0008)
	**Mediation Model 4b:** Student-teacher relationship quality (X) → Adult diet quality (M) → CMR (Y):	
+	X → Y (total effect, path c)	-0.0021 (-0.0162 to 0.0112)
++	X → M (path a)	0.1778* (0.0308 to 0.3247)
	Covariates:	
	Biological sex (female vs. male)	1.7082* (0.0729 to 3.3435)
	Black (vs. white)	0.5045 (-2.3364 to 3.3453)
	Latino (vs. white)	0.4170 (-2.7742 to 3.6082)
	'Other' (vs. white)	1.5910 (-2.0755 to 5.2576)
	Child BMI percentile	0.0123 (-0.0194 to 0.0440)
	Parental SES	3.5528*** (2.7007 to 4.4048)
+++	M → Y (path b)	-0.0139*** (-0.0210 to -0.0068)
	X → Y (direct effect, path c')	0.0004 (-0.0137 to 0.0135)
	Covariates:	
	Biological sex (female vs. male)	-0.5113*** (-0.6620 to -0.3607)
	Black (vs. white)	0.0213 (-0.2453 to 0.2878)
	Latino (vs. white)	-0.1536 (-0.4709 to 0.1638)
	'Other' (vs. white)	-0.1564 (-0.5080 to 0.1953)
	Child BMI percentile	0.0070*** (0.0041 to 0.0100)
	Parental SES	-0.1546*** (-0.2417 to -0.0675)
++++	X → M → Y (indirect effect)	-0.0025* (-0.0052 to -0.0006)
	**Mediation Model 4c:** Classroom emotional quality (X) → Adult diet quality (M) → CMR (Y):	
+	X → Y (total effect, path c)	-0.0051 (-0.1251 to 0.1141)
++	X → M (path a)	1.0081 (-0.4347 to 2.4509)
	Covariates:	
	Biological sex (female vs. male)	2.2470** (0.6829 to 3.8112)
	Black (vs. white)	0.1796 (-2.6640 to 3.0232)
	Latino (vs. white)	0.2738 (-2.9264 to 3.4739)
	'Other' (vs. white)	1.3506 (-2.3243 to 5.0255)
	Child BMI percentile	0.0099 (-0.0216 to 0.0415)

© 2025 Bleil ME et al. *JAMA Network Open*.

	Parental SES	3.6088*** (2.7511 to 4.4665)
+++	M → Y (path b)	-0.0139*** (-0.0210 to -0.0068)
	X → Y (direct effect, path c’)	0.0088 (-0.1104 to 0.1278)
	Covariates:	
	Biological sex (female vs. male)	-0.5105*** (-0.6550 to -0.3659)
	Black (vs. white)	0.0226 (-0.2414 to 0.2866)
	Latino (vs. white)	-0.1535 (-0.4702 to 0.1631)
	‘Other’ (vs. white)	-0.1573 (-0.5095 to 0.1948)
	Child BMI percentile	0.0070*** (0.0041 to 0.0100)
	Parental SES	-0.1554** (-0.2458 to -0.0650)
++++	X → M → Y (indirect effect)	-0.0139 (-0.0391 to 0.0053)
**Mediation Model 4d:** Classroom instructional quality (X) → Adult diet quality (M) → CMR (Y):		
+	X → Y (total effect, path c)	-0.0264 (-0.1506 to 0.1031)
++	X → M (path a)	0.5937 (-0.9834 to 2.1708)
	Covariates:	
	Biological sex (female vs. male)	2.2375** (0.6662 to 3.8088)
	Black (vs. white)	0.0551 (-2.7794 to 2.8900)
	Latino (vs. white)	0.2698 (-2.9347 to 3.4743)
	‘Other’ (vs. white)	1.3809 (-2.2995 to 5.0613)
	Child BMI percentile	0.0096 (-0.0220 to 0.0413)
	Parental SES	3.6942*** (2.8453 to 4.5430)
+++	M → Y (path b)	-0.0139*** (-0.0209 to -0.0068)
	X → Y (direct effect, path c’)	-0.0181 (-0.1424 to 0.1103)
	Covariates:	
	Biological sex (female vs. male)	-0.5090*** (-0.6538 to -0.3642)
	Black (vs. white)	0.0146 (-0.2487 to 0.2779)
	Latino (vs. white)	-0.1549 (-0.4707 to 0.1609)
	‘Other’ (vs. white)	-0.1553 (-0.5076 to 0.1970)
	Child BMI percentile	0.0070*** (0.0040 to 0.0100)
	Parental SES	-0.1526*** (-0.2411 to -0.0640)
++++	X → M → Y (indirect effect)	-0.0082 (-0.0360 to 0.0118)
**Mediation Model 4e:** Academic achievement (X) → Adult diet quality (M) → CMR (Y):		
+	X → Y (total effect, path c)	-0.0040 (-0.0112 to 0.0029)
++	X → M (path a)	-0.0018 (-0.0853 to 0.0816)
	Covariates:	
	Biological sex (female vs. male)	2.2790** (0.7135 to 3.8444)
	Black (vs. white)	-0.1369 (-3.0553 to 2.7816)
	Latino (vs. white)	0.2276 (-2.9883 to 3.4435)
	‘Other’ (vs. white)	1.4293 (-2.2510 to 5.1097)
	Child BMI percentile	0.0089 (-0.0228 to 0.0405)
	Parental SES	3.7601*** (2.8573 to 4.6629)
+++	M → Y (path b)	-0.0139*** (-0.0210 to -0.0068)
	X → Y (direct effect, path c’)	-0.0041 (-0.0113 to 0.0030)
	Covariates:	
	Biological sex (female vs. male)	-0.5117*** (-0.6564 to -0.3671)
	Black (vs. white)	-0.0148 (-0.2816 to 0.2521)
	Latino (vs. white)	-0.1679 (-0.4832 to 0.1475)
	‘Other’ (vs. white)	-0.1548 (-0.5059 to 0.1962)
	Child BMI percentile	0.0071*** (0.0042 to 0.0101)
	Parental SES	-0.1369** (-0.2335 to -0.0402)
++++	X → M → Y (indirect effect)	0.0000 (-0.0010 to 0.0012)

Covariates included biological sex, race/ethnicity, child BMI percentile, and parental SES.
The arrow symbol (→) denotes a path between the variables.
*<.05; **<.01; ***<.001; †<.10

© 2025 Bleil ME et al. *JAMA Network Open*.

+Y regressed onto X and covariates, excluding M (covariate effects not shown)
++M regressed onto X and covariates
+++Y regressed onto X, M, and covariates
++++Mediated effects of X on Y via M

© 2025 Bleil ME et al. *JAMA Network Open*.

eTable 5. Mediated Effects of the Child Educational Indicators on Adult CMR via Adult Educational Attainment, Adjusted for Covariates

	**Mediation Model 5a:** Student social competence (X) → Adult educational attainment (M) → CMR (Y):	**Estimate (95% CI)**
+	X → Y (total effect, path c)	-0.0079* (-0.0154 to -0.0005)
++	X → M (path a)	0.0399*** (0.0286 to 0.0512)
	Covariates:	
	Biological sex (female vs. male)	0.3298** (0.1180 to 0.5415)
	Black (vs. white)	-0.0467 (-0.4196 to 0.3263)
	Latino (vs. white)	-0.3397 (-0.7780 to 0.0987)
	‘Other’ (vs. white)	0.0956 (-0.4013 to 0.5924)
	Child BMI percentile	-0.0011 (-0.0054 to 0.0031)
	Parental SES	0.6756*** (0.5600 to 0.7911)
+++	M → Y (path b)	-0.0019 (-0.0526 to 0.0488)
	X → Y (direct effect, path c’)	-0.0078† (-0.0154 to -0.0001)
	Covariates:	
	Biological sex (female vs. male)	-0.5480*** (-0.6934 to -0.4027)
	Black (vs. white)	-0.0290 (-0.2938 to 0.2359)
	Latino (vs. white)	-0.1696 (-0.4882 to 0.1490)
	‘Other’ (vs. white)	-0.1828 (-0.5387 to 0.1732)
	Child BMI percentile	0.0066*** (0.0037 to 0.0096)
	Parental SES	-0.1816*** (-0.2719 to -0.0913)
++++	X → M → Y (indirect effect)	-0.0001 (-0.0022 to 0.0019)
	**Mediation Model 5b:** Student-teacher relationship quality (X) → Adult educational attainment (M) → CMR (Y):	
+	X → Y (total effect, path c)	-0.0021 (-0.0148 to 0.0114)
++	X → M (path a)	0.0575*** (0.0375 to 0.0774)
	Covariates:	
	Biological sex (female vs. male)	0.1098 (-0.1137 to 0.3333)
	Black (vs. white)	-0.0989 (-0.4755 to 0.2778)
	Latino (vs. white)	-0.3407 (-0.7842 to 0.1027)
	‘Other’ (vs. white)	0.1161 (-0.3865 to 0.6187)
	Child BMI percentile	-0.0014 (-0.0056 to 0.0029)
	Parental SES	0.7293*** (0.6153 to 0.8433)
+++	M → Y (path b)	-0.0137 (-0.0635 to 0.0361)
	X → Y (direct effect, path c’)	-0.0013 (-0.0142 to 0.0123)
	Covariates:	
	Biological sex (female vs. male)	-0.5335*** (-0.6852 to -0.3818)
	Black (vs. white)	0.0129 (-0.2544 to 0.2801)
	Latino (vs. white)	-0.1638 (-0.4837 to 0.1560)
	‘Other’ (vs. white)	-0.1768 (-0.5339 to 0.1803)
	Child BMI percentile	0.0069*** (0.0039 to 0.0098)
	Parental SES	-0.1940*** (-0.2832 to -0.1048)
++++	X → M → Y (indirect effect)	-0.0008 (-0.0038 to 0.0021)
	**Mediation Model 5c:** Classroom emotional quality (X) → Adult educational attainment (M) → CMR (Y):	
+	X → Y (total effect, path c)	-0.0051 (-0.1195 to 0.1110)
++	X → M (path a)	0.2283* (0.0290 to 0.4275)
	Covariates:	
	Biological sex (female vs. male)	0.2873* (0.0694 to 0.5052)
	Black (vs. white)	-0.2330 (-0.6148 to 0.1488)
	Latino (vs. white)	-0.3911† (-0.8428 to 0.0607)
	‘Other’ (vs. white)	0.0458 (-0.4663 to 0.5579)

© 2025 Bleil ME et al. *JAMA Network Open*.

	Child BMI percentile	-0.0023 (-0.0066 to 0.0021)
	Parental SES	0.7613*** (0.6437 to 0.8788)
+++	M → Y (path b)	-0.0147 (-0.0633 to 0.0340)
	X → Y (direct effect, path c’)	-0.0017 (-0.1158 to 0.1154)
	Covariates:	
	Biological sex (female vs. male)	-0.5374*** (-0.6828 to -0.3920)
	Black (vs. white)	0.0167 (-0.2482 to 0.2815)
	Latino (vs. white)	-0.1629 (-0.4826 to 0.1568)
	‘Other’ (vs. white)	-0.1754 (-0.5329 to 0.1821)
	Child BMI percentile	0.0069*** (0.0039 to 0.0098)
	Parental SES	-0.1944*** (-0.2862 to -0.1027)
++++	X → M → Y (indirect effect)	-0.0033 (-0.0220 to 0.0058)
**Mediation Model 5d:** Classroom instructional quality (X) → Adult educational attainment (M) → CMR (Y):		
+	X → Y (total effect, path c)	-0.0264 (-0.1512 to 0.1154)
++	X → M (path a)	0.4393*** (0.2247 to 0.6538)
	Covariates:	
	Biological sex (female vs. male)	0.2635* (0.0465 to 0.4805)
	Black (vs. white)	-0.1711 (-0.5507 to 0.2085)
	Latino (vs. white)	-0.3735 (-0.8212 to 0.0742)
	‘Other’ (vs. white)	0.0288 (-0.4792 to 0.5369)
	Child BMI percentile	-0.0019 (-0.0062 to 0.0024)
	Parental SES	0.7506*** (0.6355 to 0.8657)
+++	M → Y (path b)	-0.0136 (-0.0629 to 0.0357)
	X → Y (direct effect, path c’)	-0.0204 (-0.1479 to 0.1218)
	Covariates:	
	Biological sex (female vs. male)	-0.5363*** (-0.6818 to -0.3909)
	Black (vs. white)	0.0114 (-0.2530 to 0.2759)
	Latino (vs. white)	-0.1635 (-0.4824 to 0.1554)
	‘Other’ (vs. white)	-0.1740 (-0.5316 to 0.1836)
	Child BMI percentile	0.0068*** (0.0039 to 0.0098)
	Parental SES	-0.1935*** (-0.2833 to -0.1037)
++++	X → M → Y (indirect effect)	-0.0059 (-0.0328 to 0.0146)
**Mediation Model 5e:** Academic achievement (X) → Adult educational attainment (M) → CMR (Y):		
+	X → Y (total effect, path c)	-0.0040 (-0.0111 to 0.0031)
++	X → M (path a)	0.0351*** (0.0242 to 0.0460)
	Covariates:	
	Biological sex (female vs. male)	0.3084** (0.0957 to 0.5212)
	Black (vs. white)	-0.0004 (-0.3801 to 0.3793)
	Latino (vs. white)	-0.2791 (-0.7208 to 0.1625)
	‘Other’ (vs. white)	0.0465 (-0.4535 to 0.5465)
	Child BMI percentile	-0.0034 (-0.0077 to 0.0008)
	Parental SES	0.6438*** (0.5231 to 0.7645)
+++	M → Y (path b)	-0.0090 (-0.0588 to 0.0409)
	X → Y (direct effect, path c’)	-0.0037 (-0.0112 to 0.0037)
	Covariates:	
	Biological sex (female vs. male)	-0.5405*** (-0.6860 to -0.3950)
	Black (vs. white)	-0.0129 (-0.2804 to 0.2546)
	Latino (vs. white)	-0.1733 (-0.4916 to 0.1449)
	‘Other’ (vs. white)	-0.1742 (-0.5308 to 0.1825)
	Child BMI percentile	0.0070*** (0.0041 to 0.0099)
	Parental SES	-0.1833*** (-0.2779 to -0.0888)
++++	X → M → Y (indirect effect)	-0.0003 (-0.0022 to 0.0015)

Covariates included biological sex, race/ethnicity, child BMI percentile, and parental SES.
The arrow symbol (→) denotes a path between the variables.

© 2025 Bleil ME et al. *JAMA Network Open*.

*<.05; **<.01; ***<.001; †<.10
+Y regressed onto X and covariates, excluding M (covariate effects not shown)
++M regressed onto X and covariates
+++Y regressed onto X, M, and covariates
++++Mediated effects of X on Y via M

© 2025 Bleil ME et al. *JAMA Network Open*.

eTable 6. Mediated Effects of the Child Educational Indicators on Adult CMR via Adult Activity Level, Adjusted for Covariates

**Mediation Model 6a:** Student social competence (X) → Adult activity level (M) → CMR (Y):		**Estimate (95% CI)**
+	X → Y (total effect, path c)	-0.0079* (-0.0149 to -0.0001)
++	X → M (path a)	-0.446† (-0.9640 to 0.0714)
	Covariates:	
	Biological sex (female vs. male)	-5.1680 (-14.8456 to 4.5095)
	Black (vs. white)	2.1999 (-16.0141 to 20.4138)
	Latino (vs. white)	-7.8386 (-29.4543 to 13.7772)
	'Other' (vs. white)	24.8772† (-3.7067 to 53.4611)
	Child BMI percentile	0.0640 (-0.1277 to 0.2557)
	Parental SES	1.1970 (-4.3350 to 6.7290)
+++	M → Y (path b)	0.0002 (-0.0011 to 0.0014)
	X → Y (direct effect, path c')	-0.0078* (-0.0149 to 0.0000)
	Covariates:	
	Biological sex (female vs. male)	-0.5480*** (-0.6932 to -0.4028)
	Black (vs. white)	-0.0293 (-0.2945 to 0.2358)
	Latino (vs. white)	-0.1676 (-0.4847 to 0.1494)
	'Other' (vs. white)	-0.1864 (-0.5419 to 0.1691)
	Child BMI percentile	0.0066*** (0.0037 to 0.0096)
	Parental SES	-0.1832*** (-0.2674 to -0.0990)
++++	X → M → Y (indirect effect)	-0.0001 (-0.0008 to 0.0004)
**Mediation Model 6b:** Student-teacher relationship quality (X) → Adult activity level (M) → CMR (Y):		
+	X → Y (total effect, path c)	-0.0021 (-0.0149 to 0.0109)
++	X → M (path a)	-0.5478 (-1.4935 to 0.3979)
	Covariates:	
	Biological sex (female vs. male)	-3.0127 (-13.0440 to 7.0186)
	Black (vs. white)	3.1135 (-15.2724 to 21.4993)
	Latino (vs. white)	-7.7287 (-29.3302 to 13.8727)
	'Other' (vs. white)	24.7358† (-3.9376 to 53.4092)
	Child BMI percentile	0.0685 (-0.1232 to 0.2602)
	Parental SES	0.4878 (-4.8184 to 5.7941)
+++	M → Y (path b)	0.0003 (-0.0010 to 0.0015)
	X → Y (direct effect, path c')	-0.0020 (-0.0146 to 0.0112)
	Covariates:	
	Biological sex (female vs. male)	-0.5344*** (-0.6863 to -0.3826)
	Black (vs. white)	0.0133 (-0.2544 to 0.2810)
	Latino (vs. white)	-0.1573 (-0.4767 to 0.1620)
	'Other' (vs. white)	-0.1837 (-0.5401 to 0.1727)
	Child BMI percentile	0.0069*** (0.0039 to 0.0098)
	Parental SES	-0.2042*** (-0.2851 to -0.1233)
++++	X → M → Y (indirect effect)	-0.0001 (-0.0015 to 0.0004)
**Mediation Model 6c:** Classroom emotional quality (X) → Adult activity level (M) → CMR (Y):		
+	X → Y (total effect, path c)	-0.0051 (-0.1267 to 0.1174)
++	X → M (path a)	5.3698 (-3.7397 to 14.4793)
	Covariates:	
	Biological sex (female vs. male)	-4.9469 (-14.6448 to 4.7511)
	Black (vs. white)	6.6481 (-11.5379 to 24.8341)
	Latino (vs. white)	-6.9494 (-28.6434 to 14.7446)
	'Other' (vs. white)	24.8424† (-3.6437 to 53.3285)
	Child BMI percentile	0.0850 (-0.1054 to 0.2753)

© 2025 Bleil ME et al. *JAMA Network Open*.

	Parental SES	-0.8910 (-6.2855 to 4.5036)
+++	M → Y (path b)	0.0003 (-0.0010 to 0.0015)
	X → Y (direct effect, path c’)	-0.0065 (-0.1276 to 0.1155)
	Covariates:	
	Biological sex (female vs. male)	-0.5405*** (-0.6859 to -0.3952)
	Black (vs. white)	0.0182 (-0.2470 to 0.2834)
	Latino (vs. white)	-0.1554 (-0.4744 to 0.1635)
	‘Other’ (vs. white)	-0.1817 (-0.5384 to 0.1750)
	Child BMI percentile	0.0069*** (0.0039 to 0.0098)
	Parental SES	-0.2055*** (-0.2892 to -0.1217)
++++	X → M → Y (indirect effect)	0.0015 (-0.0041 to 0.0131)
**Mediation Model 6d:** Classroom instructional quality (X) → Adult activity level (M) → CMR (Y):		
+	X → Y (total effect, path c)	-0.0264 (-0.1599 to 0.1012)
++	X → M (path a)	-6.4234 (-15.9113 to 3.0645)
	Covariates:	
	Biological sex (female vs. male)	-4.3201 (-14.0667 to 5.4266)
	Black (vs. white)	3.1360 (-15.4043 to 21.6763)
	Latino (vs. white)	-7.5439 (-29.2092 to 14.1215)
	‘Other’ (vs. white)	25.7413† (-2.8566 to 54.3393)
	Child BMI percentile	0.0702 (-0.1212 to 0.2617)
	Parental SES	0.5026 (-4.7619 to 5.7672)
+++	M → Y (path b)	0.0003 (-0.0010 to 0.0015)
	X → Y (direct effect, path c’)	-0.0248 (-0.1583 to 0.1051)
	Covariates:	
	Biological sex (female vs. male)	-0.5391*** (-0.6846 to -0.3936)
	Black (vs. white)	0.0128 (-0.2518 to 0.2774)
	Latino (vs. white)	-0.1567 (-0.4746 to 0.1612)
	‘Other’ (vs. white)	-0.1798 (-0.5369 to 0.1772)
	Child BMI percentile	0.0069*** (0.0039 to 0.0098)
	Parental SES	-0.2040*** (-0.2858 to -0.1221)
++++	X → M → Y (indirect effect)	-0.0016 (-0.0150 to 0.0048)
**Mediation Model 6e:** Academic achievement (X) → Adult activity level (M) → CMR (Y):		
+	X → Y (total effect, path c)	-0.0040 (-0.0108 to 0.0032)
++	X → M (path a)	-0.6169* (-1.1071 to -0.1268)
	Covariates:	
	Biological sex (female vs. male)	-5.0182 (-14.6836 to 4.6471)
	Black (vs. white)	-0.2386 (-18.5558 to 18.0786)
	Latino (vs. white)	-9.2842 (-30.8739 to 12.3055)
	‘Other’ (vs. white)	25.5384† (-3.1117 to 54.1887)
	Child BMI percentile	0.0953 (-0.0931 to 0.2836)
	Parental SES	2.5087 (-3.0873 to 8.1047)
+++	M → Y (path b)	0.0002 (-0.0011 to 0.0015)
	X → Y (direct effect, path c’)	-0.0039 (-0.0106 to 0.0033)
	Covariates:	
	Biological sex (female vs. male)	-0.5425*** (-0.6881 to -0.3970)
	Black (vs. white)	-0.0131 (-0.2808 to 0.2547)
	Latino (vs. white)	-0.1691 (-0.4864 to 0.1482)
	‘Other’ (vs. white)	-0.1786 (-0.5346 to 0.1775)
	Child BMI percentile	0.0070*** (0.0041 to 0.0099)
	Parental SES	-0.1897*** (-0.2795 to -0.0999)
++++	X → M → Y (indirect effect)	-0.0001 (-0.0010 to 0.0005)

Covariates included biological sex, race/ethnicity, child BMI percentile, and parental SES.
The arrow symbol (→) denotes a path between the variables.
*<.05; **<.01; ***<.001; †<.10

© 2025 Bleil ME et al. *JAMA Network Open*.

+Y regressed onto X and covariates, excluding M (covariate effects not shown)
++M regressed onto X and covariates
+++Y regressed onto X, M, and covariates
++++Mediated effects of X on Y via M

© 2025 Bleil ME et al. *JAMA Network Open*.

eTable 7. Mediated Effects of the Child Educational Indicators on Adult CMR via Adult Sleep Duration, Adjusted for Covariates

**Mediation Model 7a:** Student social competence (X) → Adult sleep duration (M) → CMR (Y):		**Estimate (95% CI)**
+	X → Y (total effect, path c)	-0.0079* (-0.0159 or -0.0011)
++	X → M (path a)	-0.0009 (-0.0089 or 0.0071)
	Covariates:	
	Biological sex (female vs. male)	0.4328*** (0.2618 to 0.6038)
	Black (vs. white)	-0.5731*** (-0.8540 to -0.2923)
	Latino (vs. white)	-0.0954 (-0.4657 to 0.2749)
	'Other' (vs. white)	-0.1950 (-0.5677 to 0.1775)
	Child BMI percentile	0.0002 (-0.0032 to 0.0037)
	Parental SES	0.0660 (-0.0210 to 0.1529)
+++	M → Y (path b)	-0.0536 (-0.1391 to 0.0319)
	X → Y (direct effect, path c')	-0.0079* (-0.0158 to -0.0011)
	Covariates:	
	Biological sex (female vs. male)	-0.5252*** (-0.6745 to -0.3759)
	Black (vs. white)	-0.0592 (-0.3274 to 0.2089)
	Latino (vs. white)	-0.1730 (-0.4906 to 0.1445)
	'Other' (vs. white)	-0.1930 (-0.5493 to 0.1633)
	Child BMI percentile	0.0067*** (0.0037 to 0.0096)
	Parental SES	-0.1793*** (-0.2639 to -0.0948)
++++	X → M → Y (indirect effect)	0.0000 (-0.0004 to 0.0008)
**Mediation Model 7b:** Student-teacher relationship quality (X) → Adult sleep duration (M) → CMR (Y):		
+	X → Y (total effect, path c)	-0.0021 (-0.0143 to 0.0120)
++	X → M (path a)	-0.0011 (-0.0154 to -0.0154)
	Covariates:	
	Biological sex (female vs. male)	0.4371*** (0.2620 to 0.6122)
	Black (vs. white)	-0.5713*** (-0.8494 to -0.2931)
	Latino (vs. white)	-0.0952 (-0.4637 to 0.2733)
	'Other' (vs. white)	-0.1954 (-0.5674 to 0.1767)
	Child BMI percentile	0.0002 (-0.0033 to 0.0038)
	Parental SES	0.0646 (-0.0188 to 0.1480)
+++	M → Y (path b)	-0.0530 (-0.1389 to 0.0329)
	X → Y (direct effect, path c')	-0.0022 (-0.0143 to 0.0121)
	Covariates:	
	Biological sex (female vs. male)	-0.5116*** (-0.6676 to -0.3555)
	Black (vs. white)	-0.0158 (-0.2855 to 0.2540)
	Latino (vs. white)	-0.1631 (-0.4827 to 0.1565)
	'Other' (vs. white)	-0.1884 (-0.5458 to 0.1691)
	Child BMI percentile	0.0069*** (0.0039 to 0.0098)
	Parental SES	-0.2005*** (-0.2819 to -0.1191)
++++	X → M → Y (indirect effect)	0.0001 (-0.0008 to 0.0012)
**Mediation Model 7c:** Classroom emotional quality (X) → Adult sleep duration (M) → CMR (Y):		
+	X → Y (total effect, path c)	-0.0051 (-0.1370 to 0.1108)
++	X → M (path a)	0.0051 (-0.1466 to 0.1567)
	Covariates:	
	Biological sex (female vs. male)	0.4333*** (0.2631 to 0.6035)
	Black (vs. white)	-0.5660*** (-0.8448 to -0.2873)
	Latino (vs. white)	-0.0939 (-0.4637 to 0.2759)
	'Other' (vs. white)	-0.1948 (-0.5671 to 0.1775)

© 2025 Bleil ME et al. *JAMA Network Open*.

	Child BMI percentile	0.0003 (-0.0032 to 0.0037)
	Parental SES	0.0626 (-0.0194 to 0.1446)
+++	M → Y (path b)	-0.0530 (-0.1388 to 0.0328)
	X → Y (direct effect, path c')	-0.0045 (-0.1375 to 0.1110)
	Covariates:	
	Biological sex (female vs. male)	-0.5184*** (-0.6682 to -0.3687)
	Black (vs. white)	-0.0095 (-0.2770 to 0.2579)
	Latino (vs. white)	-0.1611 (-0.4802 to 0.1581)
	'Other' (vs. white)	-0.1860 (-0.5440 to 0.1720)
	Child BMI percentile	0.0069*** (0.0040 to 0.0099)
	Parental SES	-0.2023*** (-0.2866 to -0.1180)
++++	X → M → Y (indirect effect)	-0.0006 (-0.0114 to 0.0099)
**Mediation Model 7d:**<br>Classroom instructional quality (X) → Adult sleep duration (M) → CMR (Y):		
+	X → Y (total effect, path c)	-0.0264 (-0.1529 to 0.1063)
++	X → M (path a)	-0.0217 (-0.1744 to 0.1310)
	Covariates:	
	Biological sex (female vs. male)	0.4351*** (0.2645 to 0.6057)
	Black (vs. white)	-0.5739*** (-0.8556 to -0.2922)
	Latino (vs. white)	-0.0953 (-0.4648 to 0.2741)
	'Other' (vs. white)	-0.1927 (-0.5653 to 0.1800)
	Child BMI percentile	0.0002 (-0.0032 to 0.0037)
	Parental SES	0.0655 (-0.0181 to 0.1491)
+++	M → Y (path b)	-0.0531 (-0.1389 to 0.0326)
	X → Y (direct effect, path c')	-0.0272 (-0.1534 to 0.1062)
	Covariates:	
	Biological sex (female vs. male)	-0.5166*** (-0.6666 to -0.3666)
	Black (vs. white)	-0.0163 (-0.2836 to 0.2509)
	Latino (vs. white)	-0.1624 (-0.4806 to 0.1558)
	'Other' (vs. white)	-0.1842 (-0.5422 to 0.1738)
	Child BMI percentile	0.0069*** (0.0039 to 0.0099)
	Parental SES	-0.2003*** (-0.2826 to -0.1179)
++++	X → M → Y (indirect effect)	0.0009 (-0.0084 to 0.0141)
**Mediation Model 7e:**<br>Academic achievement (X) → Adult sleep duration (M) → CMR (Y):		
+	X → Y (total effect, path c)	-0.0040 (-0.0110 to 0.0031)
++	X → M (path a)	-0.0023 (-0.0103 to 0.0057)
	Covariates:	
	Biological sex (female vs. male)	0.4327*** (0.2621 to 0.6032)
	Black (vs. white)	-0.5871*** (-0.8717 to -0.3026)
	Latino (vs. white)	-0.1020 (-0.4713 to 0.2673)
	'Other' (vs. white)	-0.1931 (-0.5652 to 0.1790)
	Child BMI percentile	0.0003 (-0.0032 to 0.0038)
	Parental SES	0.0731 (-0.0194 to 0.1657)
+++	M → Y (path b)	-0.0539 (-0.1401 to 0.0323)
	X → Y (direct effect, path c')	-0.0042 (-0.0111 to 0.0030)
	Covariates:	
	Biological sex (female vs. male)	-0.5197*** (-0.6697 to -0.3698)
	Black (vs. white)	-0.0443 (-0.3151 to 0.2264)
	Latino (vs. white)	-0.1753 (-0.4929 to 0.1423)
	'Other' (vs. white)	-0.1846 (-0.5416 to 0.1724)
	Child BMI percentile	0.0070*** (0.0041 to 0.0100)
	Parental SES	-0.1851*** (-0.2750 to -0.0951)
++++	X → M → Y (indirect effect)	0.0001 (-0.0002 to 0.0010)

Covariates included biological sex, race/ethnicity, child BMI percentile, and parental SES.
The arrow symbol (→) denotes a path between the variables.

© 2025 Bleil ME et al. *JAMA Network Open*.

*<.05; **<.01; ***<.001; †<.10
+Y regressed onto X and covariates, excluding M (covariate effects not shown)
++M regressed onto X and covariates
+++Y regressed onto X, M, and covariates
++++Mediated effects of X on Y via M

© 2025 Bleil ME et al. *JAMA Network Open*.

eTable 8. Mediated Effects of the Child Educational Indicators on Adult CMR via Adult Smoking Status, Adjusted for Covariates

**Mediation Model 8a:** Student social competence (X) → Adult smoking status (M) → CMR (Y):		**Estimate (95% CI)**
+	X → Y (total effect, path c)	-0.0079* (-0.0153 to 0.0001)
	X → M (path a)	-0.0335*** (0.9493 to 0.9853)
	Covariates:	
	Biological sex (female vs. male)	-0.9106*** (0.2821 to 0.5737)
++	Black (vs. white)	-0.8077* (0.2352 to 0.8454)
	Latino (vs. white)	-0.4849 (0.2937 to 1.2908)
	‘Other’ (vs. white)	-0.3275 (0.3125 to 1.6622)
	Child BMI percentile	0.0073* (1.0000 to 1.0147)
	Parental SES	-0.3072** (0.5990 to 0.9032)
	M → Y (path b)	0.0425 (-0.1239 to 0.2089)
	X → Y (direct effect, path c’)	-0.0076† (-0.0150 to 0.0006)
	Covariates:	
	Biological sex (female vs. male)	-0.5414*** (-0.6900 to -0.3927)
+++	Black (vs. white)	-0.0225 (-0.2876 to 0.2427)
	Latino (vs. white)	-0.1652 (-0.4825 to 0.1521)
	‘Other’ (vs. white)	-0.1804 (-0.5354 to 0.1745)
	Child BMI percentile	0.0066*** (0.0036 to 0.0096)
	Parental SES	-0.1806*** (-0.2654 to -0.0958)
++++	X → M → Y (indirect effect)	-0.0003 (-0.0016 to 0.0009)
**Mediation Model 8b:** Student-teacher relationship quality (X) → Adult smoking status (M) → CMR (Y):		
+	X → Y (total effect, path c)	-0.0021 (-0.0146 to 0.0122)
	X → M (path a)	-0.0406* (0.9306 to 0.9908)
	Covariates:	
	Biological sex (female vs. male)	-0.7378*** (0.3326 to 0.6875)
++	Black (vs. white)	-0.7394* (0.2525 to 0.9026)
	Latino (vs. white)	-0.4904 (0.2913 to 1.2876)
	‘Other’ (vs. white)	-0.3234 (0.3167 to 1.6537)
	Child BMI percentile	0.0076* (1.0004 to 1.0150)
	Parental SES	-0.3656*** (0.5676 to 0.8480)
	M → Y (path b)	0.0637 (-0.1023 to 0.2296)
	X → Y (direct effect, path c’)	-0.0016 (-0.0139 to 0.0126)
	Covariates:	
	Biological sex (female vs. male)	-0.5261*** (-0.6802 to -0.3720)
+++	Black (vs. white)	0.0230 (-0.2445 to 0.2905)
	Latino (vs. white)	-0.1535 (-0.4733 to 0.1663)
	‘Other’ (vs. white)	-0.1745 (-0.5306 to 0.1816)
	Child BMI percentile	0.0068*** (0.0038 to 0.0098)
	Parental SES	-0.1999*** (-0.2817 to -0.1180)
++++	X → M → Y (indirect effect)	-0.0005 (-0.0025 to 0.0007)
**Mediation Model 8c:** Classroom emotional quality (X) → Adult smoking status (M) → CMR (Y):		
+	X → Y (total effect, path c)	-0.0051 (-0.1156 to 0.1129)
	X → M (path a)	-0.0572 (0.6951 to 1.2831)
	Covariates:	
	Biological sex (female vs. male)	-0.8586*** (0.2987 to 0.6012)
++	Black (vs. white)	-0.5969† (0.2951 to 1.0269)
	Latino (vs. white)	-0.4548 (0.3028 to 1.3298)
	‘Other’ (vs. white)	-0.2699 (0.3366 to 1.7316)
	Child BMI percentile	0.0082* (1.0010 to 1.0155)

© 2025 Bleil ME et al. *JAMA Network Open*.

	Parental SES	-0.4072*** (0.5437 to 0.8146)
+++	M → Y (path b)	0.0655 (-0.0992 to 0.2303)
	X → Y (direct effect, path c’)	-0.0044 (-0.1160 to 0.1125)
	Covariates:	
	Biological sex (female vs. male)	-0.5308*** (-0.6794 to -0.3822)
	Black (vs. white)	0.0275 (-0.2370 to 0.2920)
	Latino (vs. white)	-0.1519 (-0.4711 to 0.1674)
	‘Other’ (vs. white)	-0.1726 (-0.5293 to 0.1841)
	Child BMI percentile	0.0068*** (0.0038 to 0.0098)
	Parental SES	-0.2009*** (-0.2858 to -0.1159)
++++	X → M → Y (indirect effect)	-0.0007 (-0.0110 to 0.0041)
**Mediation Model 8d:** Classroom instructional quality (X) → Adult smoking status (M) → CMR (Y):		
+	X → Y (total effect, path c)	-0.0266 (-0.1583 to 0.1118)
++	X → M (path a)	-0.4186* (0.4636 to 0.9338)
	Covariates:	
	Biological sex (female vs. male)	-0.8424*** (0.3031 to 0.6119)
	Black (vs. white)	-0.7039* (0.2639 to 0.9273)
	Latino (vs. white)	-0.4766 (0.2940 to 1.3111)
	‘Other’ (vs. white)	-0.2462 (0.3436 to 1.7789)
	Child BMI percentile	0.0077* (1.0005 to 1.0150)
	Parental SES	-0.3745*** (0.5634 to 0.8392)
+++	M → Y (path b)	0.0634 (-0.1022 to 0.2291)
	X → Y (direct effect, path c’)	-0.0215 (-0.1531 to 0.1202)
	Covariates:	
	Biological sex (female vs. male)	-0.5297*** (-0.6784 to -0.3811)
	Black (vs. white)	0.0222 (-0.2422 to 0.2865)
	Latino (vs. white)	-0.1531 (-0.4713 to 0.1652)
	‘Other’ (vs. white)	-0.1714 (-0.5282 to 0.1855)
	Child BMI percentile	0.0068*** (0.0038 to 0.0098)
	Parental SES	-0.1995*** (-0.2824 to -0.1167)
++++	X → M → Y (indirect effect)	-0.0051 (-0.0272 to 0.0065)
**Mediation Model 8e:** Academic achievement (X) → Adult smoking status (M) → CMR (Y):		
+	X → Y (total effect, path c)	-0.0040 (-0.0112 to 0.0031)
++	X → M (path a)	-0.0025 (0.9800 to 1.0155)
	Covariates:	
	Biological sex (female vs. male)	-0.8622*** (0.2976 to 0.5991)
	Black (vs. white)	-0.6025† (0.2898 to 1.0341)
	Latino (vs. white)	-0.4621 (0.3001 to 1.3223)
	‘Other’ (vs. white)	-0.2723 (0.3358 to 1.7273)
	Child BMI percentile	0.0083* (1.0011 to 1.0156)
	Parental SES	-0.4030*** (0.5389 to 0.8288)
+++	M → Y (path b)	0.0644 (-0.1001 to 0.2289)
	X → Y (direct effect, path c’)	-0.0040 (-0.0111 to 0.0031)
	Covariates:	
	Biological sex (female vs. male)	-0.5326*** (-0.6813 to -0.3839)
	Black (vs. white)	-0.0055 (-0.2730 to 0.2620)
	Latino (vs. white)	-0.1655 (-0.4832 to 0.1522)
	‘Other’ (vs. white)	-0.1712 (-0.5269 to 0.1845)
	Child BMI percentile	0.0069*** (0.0040 to 0.0099)
	Parental SES	-0.1845*** (-0.2751 to -0.0940)
++++	X → M → Y (indirect effect)	-0.0000 (-0.0007 to 0.0002)

Covariates included biological sex, race/ethnicity, child BMI percentile, and parental SES.
The arrow symbol (→) denotes a path between the variables.
*<.05; **<.01; ***<.001; †<.10

© 2025 Bleil ME et al. *JAMA Network Open*.

+Y regressed onto X and covariates, excluding M (covariate effects not shown)
++M regressed onto X and covariates
+++Y regressed onto X, M, and covariates
++++Mediated effects of X on Y via M

© 2025 Bleil ME et al. *JAMA Network Open*.
