# Supplementary material for: Mediators of the Association Between Child Education and Adult Health
Source: JAMA Netw Open. 2025 May 23;8(5):e258855. doi: 10.1001/jamanetworkopen.2025.8855 (PMC12102707; doi:10.1001/jamanetworkopen.2025.8855)
Supplement: Supplement 2. — Data Sharing Statement [file jamanetwopen-e258855-s002.pdf]

Data Sharing Statement

Bleil. Mediators of the Association Between Child Education and Adult Health. *JAMA Netw Open*. Published May 23, 2025. doi:10.1001/jamanetworkopen.2025.8855

Data
Data available: No

Additional Information
Explanation for why data not available: Data Sharing Statement: Data from the original NICHD SECCYD analyzed in the current study are available online: ICPSR NICHD Study of Early Child Care and Youth Development (SECCYD) Series https://www.icpsr.umich.edu//web/ICPSR/series/233. Data from the SHINE follow-up study analyzed in the current study are not available online due to privacy and ethical restrictions. Researchers interested in working with the team of investigators who led the SHINE follow-up data collection are invited to contact MEB and GIR. Collaborative efforts will be considered under specific conditions, including the scope of work and assurances related to data security and integrity.
